# CD112 Lactylation Drives Dual Immune Evasion From CD8+ T Cells and NK Cells

**DOI:** 10.1002/advs.78088

**Published:** 2026-10-04

**Authors:** Zhuoshuo Xu, Xinyu Zhang, Fang Dong, Nenghao Shen, Jun Zhou, Zihan Xi, Zhenghao Wu

**Affiliations:** ^1^ Department of Breast and Thyroid Surgery Union Hospital Tongji Medical College Huazhong University of Science and Technology Wuhan China; ^2^ TaiKang Medical School (School of Basic Medical Sciences) Taikang Center for Life and Medical Sciences Wuhan University Wuhan China; ^3^ Guangdong Provincial Key Laboratory of Malignant Tumor Epigenetics and Gene Regulation Medical Research Center Sun Yat‐Sen Memorial Hospital Sun Yat‐Sen University Guangzhou China

**Keywords:** AARS2, CD112 lactylation, CD8+ T cells, immunotherapy, natural killer cells, tumor immune evasion

## Abstract

Current cancer immunotherapy predominantly targets CD8+ T cells, leaving NK cell surveillance largely unaddressed. We report that lactylation of the membrane checkpoint ligand CD112 serves as a convergent mechanism for dual immune escape. In contrast to T cell–centric checkpoints such as PD‐L1, CD112 functions as a shared inhibitory ligand for both CD8+ T cells and NK cells through TIGIT/CD112R signaling. Mechanistically, the lactyltransferase AARS2 catalyzes CD112 lactylation at lysine 412 in response to tumor lactate accumulation, competitively blocking the HECT E3 ligase UBE3C to suppress K48/K63‐linked ubiquitination and lysosomal degradation. This stabilizes CD112 at the plasma membrane, enabling tumor cells to coordinately evade both adaptive and innate cytotoxic lymphocytes. Disrupting this axis restores dual immune surveillance and suppresses tumor growth. Tumors with high CD112 lactylation and surface enrichment are selectively vulnerable to TIGIT/PD‐L1 bispecific antibody blockade and HSV‐2 oncolytic virotherapy, nominating CD112 lactylation status as a predictive biomarker for combination immunotherapy.

## Graphical abstract

AARS2 functions as an intracellular lactate sensor and lactyltransferase, catalyzing CD112 K412 lactylation to block ubiquitination‐dependent degradation and stabilize CD112. This pathway suppresses CD8+ T‐cell and NK‐cell cytotoxicity, enabling dual immune evasion. CD112 K412 lactylation further stratifies responses to dual checkpoint blockade and oncolytic virotherapy, revealing a potential biomarker and therapeutic target.

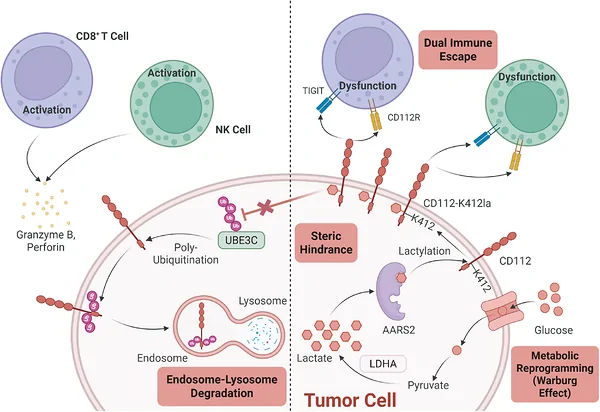

## 1 Introduction

Immune evasion is a hallmark of malignant progression. The human antitumor immune system relies on a dual surveillance network comprising CD8^+^ cytotoxic T lymphocytes (CTLs) and natural killer (NK) cells, which provide precise, antigen‐specific killing and broad, innate immunosurveillance, respectively [1, 2, 3]. Although immune checkpoint blockade (ICB) targeting the PD‐1/PD‐L1 axis has revolutionized cancer therapy, clinical response rates remain limited to 20%–30%, and a fundamental bottleneck is that current strategies exclusively target CTLs while failing to restore NK cell‐mediated cytotoxicity or prevent tumor escape from dual immune surveillance [4, 5, 6]. Notably, tumors can simultaneously evade both CTL and NK cell killing through shared molecular mechanisms, suggesting the existence of a convergent regulatory node that orchestrates dual immune evasion [7, 8, 9]. Therefore, elucidating the molecular mechanism by which tumors synchronously escape surveillance by both CD8+ T cells and NK cells represents a critical scientific question for overcoming the efficacy ceiling of current immunotherapies.

Tumor metabolic reprogramming, characterized by aerobic glycolysis (the Warburg effect), drives massive lactate accumulation in the tumor microenvironment (TME) [10, 11, 12]. Beyond its classical role as a metabolic waste product, lactate functions as a signaling molecule that acidifies the TME and directly suppresses T and NK cell activation, thereby fostering an immunosuppressive niche [13, 14, 15]. Recently, lactate has been identified as a direct chemical substrate for protein lactylation, a newly discovered post‐translational modification that regulates non‐histone protein function [16, 17, 18]. AARS1 and AARS2 were subsequently characterized as intracellular L‐lactate sensors and lactyltransferases that catalyze lysine lactylation in an ATP‐dependent manner [19, 20]. While AARS1 primarily promotes tumor cell proliferation through cell‐intrinsic mechanisms, the immune‐regulatory function of AARS2—particularly whether it links tumor lactate metabolism to immune evasion—remains poorly defined.

This study aims to elucidate how AARS2‐mediated lactyltransferase activity functions as a metabolic–immunological coupling mechanism to orchestrate tumor immune evasion. Specifically, we seek to identify the critical downstream substrates and molecular pathways through which AARS2 enables tumors to synchronously escape surveillance by both CD8+ T cells and NK cells.

## 2 Results

### 2.1 CRISPR Screens Identify AARS2 as a Candidate Gene for Dual Tumor Immune Escape

To identify genes that enable tumor cells to escape immune killing by both CD8+ T cells and NK cells, we integrated three independent CRISPR functional screening datasets. The overall screening strategy included in vivo immune pressure screens, antigen‐specific CD8+ T cell killing screens, and NK cell killing screens. This design first allowed us to select candidate genes linked to tumor immune adaptation in an in vivo immune setting and then to determine whether these genes affected CD8+ T cell‐ and NK cell‐mediated cytotoxicity.

First, to distinguish general tumor growth dependency genes from immune pressure‐associated genes, we analyzed in vivo CRISPR screening data. 4T1 breast cancer cells and CT26 colon cancer cells carrying a mouse druggable CRISPR library were screened under three conditions: in vitro culture, immunocompetent mice, and immunodeficient mice. This library contained approximately 38 000 sgRNAs targeting about 7500 genes. Tumor cells from each condition were collected, and changes in the abundance of gene knockout clones were measured by sgRNA sequencing [21]. (Figure 1a). By comparing sgRNA enrichment between immunocompetent and immunodeficient mice, MAGeCK identified 254 commonly and significantly enriched genes (*p* < 0.05). *Aars2* appeared in the significant candidate region together with Mars2, Ahcy, Mthfd1, Hsd17b10, and Pgs1, matching the labeled hits in Figure 1b. These genes were defined as required for maintaining tumor growth in the presence of functional CD8+ T cells and NK cells, suggesting that they may take part in tumor immune escape (Figure 1b).

**FIGURE 1:**
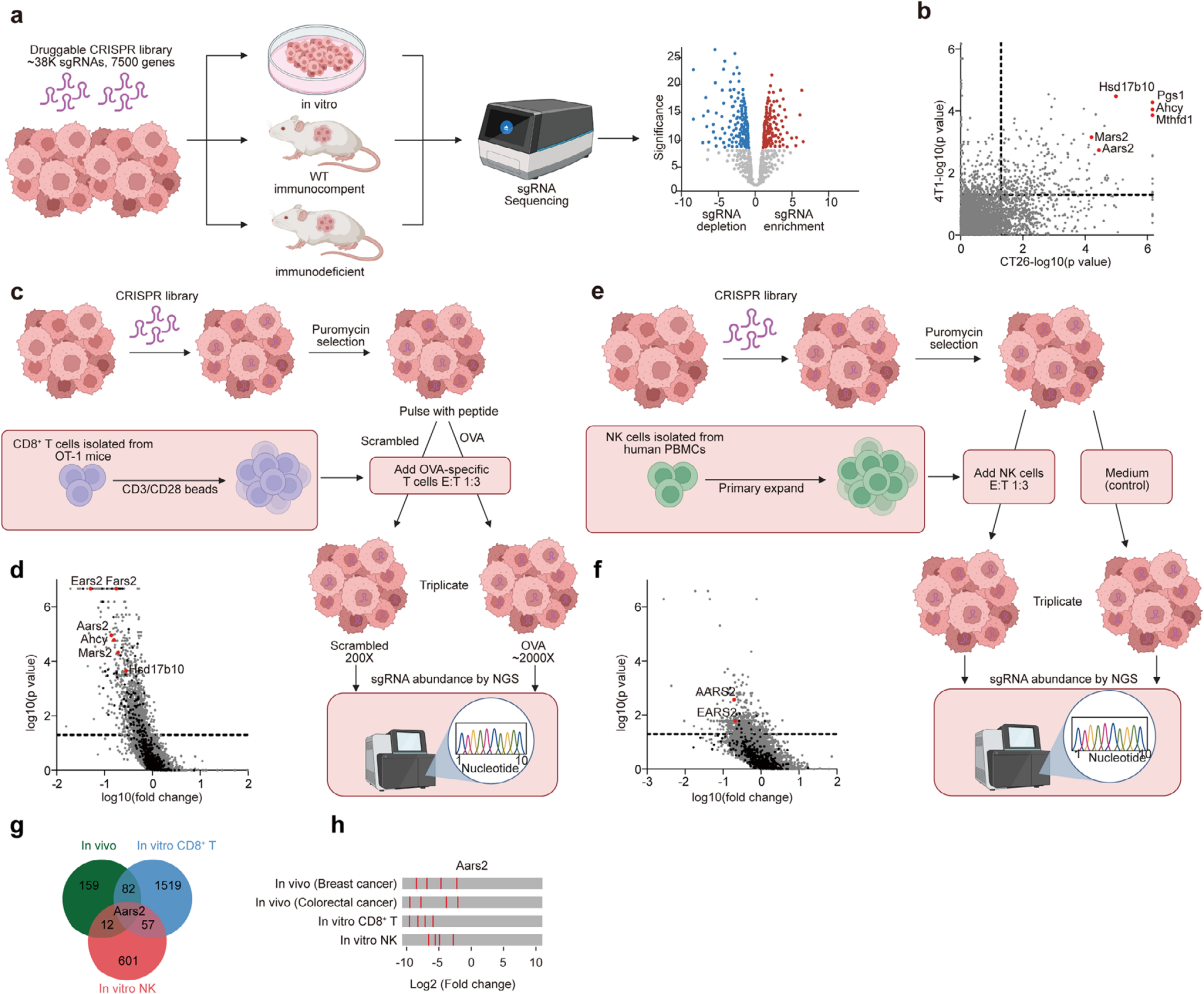
CRISPR screens identify AARS2 as a common regulator of tumor escape from CD8+ T cell and NK cell surveillance (a) Schematic illustration of the in vivo immune‐pressure CRISPR screening strategy. 4T1 breast cancer cells and CT26 colorectal cancer cells carrying a druggable CRISPR knockout library were maintained in vitro or subcutaneously transplanted into immunocompetent wild‐type mice and immunodeficient mice. Tumor cells were harvested after tumor growth, and sgRNA abundance was quantified by next‐generation sequencing (NGS). (b) Scatter plot showing genes enriched under immune‐competent versus immune‐deficient conditions in the 4T1 and CT26 in vivo screens. Dashed lines indicate the significance cutoff. *Aars2*, together with *Mars2*, *Ahcy*, *Mthfd1*, *Hsd17b10*, and *Pgs1*, was identified among the shared significant candidates, suggesting a role in tumor growth under functional immune pressure. (c) Schematic of the antigen‐specific CD8+ T cell killing CRISPR screen. Tumor cells carrying the CRISPR library were selected with puromycin, pulsed with OVA peptide or scrambled control peptide, and then cocultured with activated OT‐I CD8+ T cells at an effector‐to‐target ratio of 1:3. Surviving tumor cells were collected in triplicate, and sgRNA abundance was analyzed by NGS. (d) Volcano plot of the antigen‐specific CD8+ T cell killing screen. Genes whose sgRNAs were depleted in surviving OVA‐pulsed tumor cells were identified as candidate mediators of resistance to CD8+ T cell cytotoxicity. *Aars2* was among the significant hits, together with *Ears2*, *Fars2*, *Ahcy*, *Mars2* and *Hsd17b10*. (e) Schematic of the NK cell cytotoxicity CRISPR screen. Tumor cells carrying the CRISPR knockout library were selected with puromycin and then cocultured with ex vivo expanded primary human NK cells at an effector‐to‐target ratio of 1:3, with medium‐only culture as the control. Surviving tumor cells were collected in triplicate for NGS‐based sgRNA abundance analysis. (f) Volcano plot of the NK cell cytotoxicity screen. Genes whose sgRNAs were depleted after NK cell co‐culture were defined as candidate genes required for tumor cell resistance to NK cell‐mediated killing. *AARS2* was identified as a significant negatively selected hit. (g) Venn diagram showing the intersection of candidates from the in vivo immune‐pressure screen, the antigen‐specific CD8+ T cell killing screen and the NK cell cytotoxicity screen. *AARS2* was the only shared candidate across all three screens. (h) Distribution of *Aars2*‐targeting sgRNAs across the four screening datasets, including in vivo breast cancer, in vivo colorectal cancer, in vitro CD8+ T cell killing and in vitro NK cell killing screens. The consistent negative shifts of *Aars2*‐targeting sgRNAs support AARS2 as a common regulator of tumor resistance to both CD8+ T cell and NK cell immune pressure.

Because in vivo screening can identify immune escape‐associated genes but cannot distinguish whether they mediate escape from CD8+ T cells or from NK cells, and because other immune components such as macrophages and dendritic cells may also contribute, we next used an antigen‐specific CD8+ T cell killing screen to define the specific role of candidate genes in T cell immune escape. In this system, mouse tumor cells carrying the sgRNA library were pulsed with MHC‐I‐restricted ovalbumin (OVA) peptide or a scrambled control peptide and then cocultured for 24 h with activated CD8+ T cells isolated from the spleens of OT‐I transgenic mice, whose T cell receptors specifically recognize OVA peptide. Surviving tumor cells were then sorted by flow cytometry, and sgRNA abundance was quantified [22] (Figure 1c). Among the candidate genes identified from the in vivo immune‐pressure screens, 1659 genes showed significant changes in the antigen‐specific CD8+ T‐cell killing screen. In this negative‐selection screen, sgRNA abundance in surviving tumor cells from the OVA‐specific CD8+ T‐cell group was compared with that in the scrambled‐peptide control group. sgRNAs targeting Ears2, Fars2, Aars2, Ahcy, Mars2, and Hsd17b10 were markedly depleted in the OVA group. Because sgRNA abundance was quantified in the surviving tumor‐cell population, this depletion indicates that tumor‐cell clones carrying knockout of these genes were preferentially eliminated under antigen‐specific CD8+ T‐cell pressure. Thus, loss of these genes sensitized tumor cells to CD8+ T‐cell‐mediated killing, suggesting that the corresponding intact genes support tumor‐cell survival and resistance under immune pressure (Figure 1d).

To further identify genes that mediate NK cell escape, we analyzed CRISPR screening data from an NK cytotoxicity screen. In this screen, tumor cells carrying a CRISPR knockout library were selected to generate a stable library and then cocultured with in vitro‐expanded human peripheral blood‐derived NK cells or maintained in regular medium as a control. After co‐culture, surviving tumor cells were collected and subjected to sgRNA abundance sequencing to assess how each gene knockout affected tumor cell survival under NK cell killing pressure [23] (Figure 1e). A total of 671 genes were consistently identified as negatively selected in the NK‐cell killing screen. Specifically, their corresponding sgRNAs were less abundant in surviving tumor cells from the NK‐cell co‐culture group than in those from the medium‐only control group. This depletion indicates that disruption of these genes reduced tumor‐cell survival under NK‐cell pressure and increased susceptibility to NK‐cell‐mediated killing. These genes were therefore considered candidate factors that support tumor‐cell resistance to NK‐cell immune surveillance (Figure 1f).

Cross‐analysis of these three screening datasets identified *AARS2* as a shared candidate gene that not only mediates tumor adaptation to the adaptive immune system in vivo but also enables cancer cells to evade antigen‐specific CD8+ T cell and NK cell killing in vitro (Figure 1g,h).

### 2.2 AARS2 Promotes Tumor Growth by Suppressing CD8+ T Cell and NK Cell Immune Surveillance

To define the functional differences between AARS1 and AARS2 during tumor progression, we knocked out *Aars1* or *Aars2* in multiple mouse tumor cell lines, including EO771 breast cancer, Panc02 pancreatic ductal cancer, and B16‐F10 melanoma cells, and implanted these cells subcutaneously into C57BL/6 mice. In the B16‐F10 melanoma model, loss of either *Aars1* or *Aars2* reduced endpoint tumor weight and prolonged mouse survival, with a stronger antitumor effect after *Aars2* loss (Figure 2a,b; Figure S1a,b). In the EO771 breast cancer model, *Aars2* loss significantly suppressed tumor growth, reduced endpoint tumor weight, and prolonged survival, whereas *Aars1* loss did not produce a stable antitumor effect (Figure 2c,d and Figure S1c,d). Similarly, in the Panc02 pancreatic ductal cancer model, *Aars2* knockout also slowed tumor growth and improved survival, whereas *Aars1* knockout did not differ significantly from the control group (Figure S1e–h). These findings indicate that Aars2 has a more consistent role in sustaining in vivo growth across different mouse tumor models. Although previous work has shown that AARS1 promotes tumorigenesis by lactylating p53, the cancer‐promoting mechanism of AARS2 remains unclear [20]. To further confirm the tumor‐promoting role of Aars2, we reintroduced *Aars2* cDNA into *Aars2*‐deficient EO771 cells. *Aars2* re‐expression markedly restored tumor growth capacity, as shown by increased tumor volume, higher endpoint tumor weight, and shorter mouse survival (Figure 2e,f and Figure S1i,j). This rescue experiment reduces the possibility that the phenotype was caused by sgRNA off‐target effects and further supports Aars2 as a functional driver of in vivo tumor growth.

**FIGURE 2:**
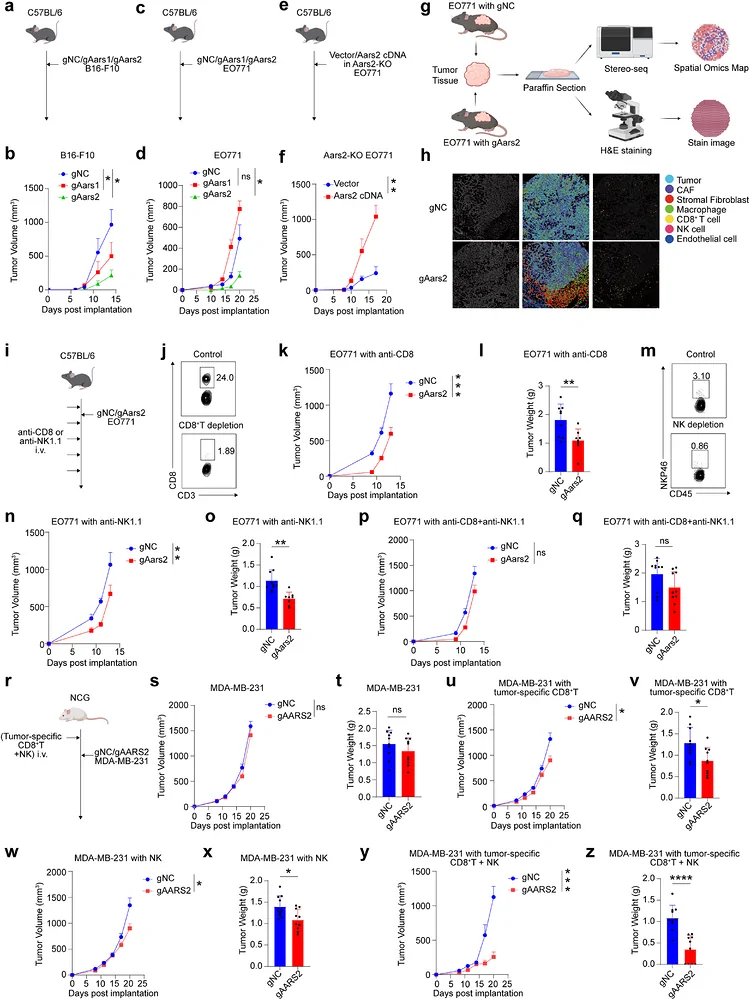
AARS2 promotes tumor progression by suppressing CD8+ T cell and NK cell‐mediated antitumor immunity (a–f) *Aars2* deficiency suppresses tumor growth in immunocompetent C57BL/6 mice, and *Aars2* re‐expression restores the growth of *Aars2*‐knockout EO771 tumors. (a) Schematic of the subcutaneous B16‐F10 tumor model using control, *Aars1*‐knockout, or Aars2‐knockout cells. (b) Tumor growth curves of subcutaneous B16‐F10 tumors. *n* = 6–14 mice per group, *p = 0.0303. (c) Schematic of the subcutaneous EO771 tumor model using control, *Aars1*‐knockout, or *Aars2*‐knockout cells. (d) Tumor growth curves of subcutaneous EO771 tumors. *n* = 6–14 mice per group, *p = 0.0195. (e) Schematic of *Aars2*‐knockout EO771 tumors reconstituted with empty vector or *Aars2* cDNA before subcutaneous implantation. (f) Tumor growth curves of reconstituted *Aars2*‐knockout EO771 tumors. *n* = 6–14 mice per group, ^**^ *p* = 0.0083. (g) Experimental workflow for Stereo‐seq spatial transcriptomic analysis and H&E staining of control and *Aars2*‐knockout EO771 tumors. (h) Representative spatial maps showing the distribution of major cell populations in control and *Aars2*‐knockout EO771 tumors, including tumor cells, cancer‐associated fibroblasts (CAFs), stromal fibroblasts, macrophages, CD8+ T cells, NK cells, and endothelial cells. (i) Schematic of in vivo immune cell depletion experiments in the EO771 model. Control or *Aars2*‐knockout EO771 cells were implanted into C57BL/6 mice, followed by treatment with anti‐CD8, anti‐NK1.1, or combined anti‐CD8 plus anti‐NK1.1 antibodies. (j) Representative flow cytometry plots confirming CD8+ T cell depletion after anti‐CD8 treatment. k,l, Tumor growth curves, ***p = 0.0004. (k) and endpoint tumor weights, **p = 0.0093. (l) of control and *Aars2*‐knockout EO771 tumors after CD8+ T cell depletion. *Aars2* knockout still reduced tumor growth and tumor weight under CD8+ T cell depletion. *n* = 6–14 mice per group. (m) Representative flow cytometry plots confirming NK cell depletion after anti‐NK1.1 treatment. n,o, Tumor growth curves, **p = 0.0049. (n) and endpoint tumor weights, **p = 0.001. (o) of control and *Aars2*‐knockout EO771 tumors after NK cell depletion. *Aars2* knockout still reduced tumor growth and tumor weight under NK cell depletion. *n* = 6–14 mice per group. (p, q) Tumor growth curves (p) and endpoint tumor weights (q) of control and *Aars2*‐knockout EO771 tumors after combined CD8+ T cell and NK cell depletion. The growth difference between control and *Aars2*‐knockout tumors was largely lost after simultaneous depletion of both cell populations. *n* = 6–14 mice per group. (r) Schematic of the human tumor immune reconstitution model. Control or *AARS2*‐knockout MDA‐MB‐231 cells were implanted into NCG mice, followed by adoptive transfer of tumor‐specific CD8+ T cells, NK cells, or both cell types. s,t, Tumor growth curves (s) and endpoint tumor weights (t) of control and *AARS2*‐knockout MDA‐MB‐231 tumors in NCG mice without immune cell transfer. *AARS2* knockout did not significantly affect tumor growth in the absence of transferred immune cells. *n* = 6–14 mice per group. (u, v) Tumor growth curves, *p = 0.0173. (u) and endpoint tumor weights, *p = 0.0146. (v) of control and *AARS2*‐knockout MDA‐MB‐231 tumors after transfer of tumor‐specific CD8+ T cells. *AARS2* knockout increased the antitumor effect of CD8+ T cells. *n* = 6–14 mice per group. w,x, Tumor growth curves, *p = 0.0299. (w) and endpoint tumor weights, *p = 0.0122. (x) of control and *AARS2*‐knockout MDA‐MB‐231 tumors after transfer of NK cells. *AARS2* knockout increased the antitumor effect of NK cells. *n* = 6–14 mice per group. (y, z) Tumor growth curves, ***p = 0.0003. (y) and endpoint tumor weights, ****p < 0.0001. (z) of control and *AARS2*‐knockout MDA‐MB‐231 tumors after combined transfer of tumor‐specific CD8+ T cells and NK cells. Combined immune cell transfer produced the strongest suppression of *AARS2*‐knockout tumors. *n* = 6–14 mice per group. Data are presented as mean ± s.d. Tumor growth curves were analyzed by two‐way ANOVA with a multiple‐comparisons test. Endpoint tumor weights were analyzed by a two‐tailed unpaired *t*‐test. *p < 0.05, **p < 0.01, ^***^ *p* < 0.001, ****p < 0.0001; ns, not significant.

To determine whether Aars2 acts through the tumor immune microenvironment, we used Stereo‐seq spatial transcriptomics to analyze cell composition and spatial distribution in EO771 control and *Aars2*‐deficient tumors (Figure 2g). Using the Stereo‐seq platform, we captured 16 930 high‐quality cells. UMAP dimensionality reduction and clustering identified 29 transcriptionally homogeneous cell clusters that separated control and *Aars2*‐knockout tumors. These clusters were further annotated as major cell types, including tumor cells, cancer‐associated fibroblasts (CAFs), fibroblasts, macrophages, CD8+ T cells, NK cells, and endothelial cells. SingleR annotation and marker gene expression further defined the spatial distribution patterns of these cell subsets in tumor tissues (Figure S1k–m). Spatial maps showed clear changes in immune cell composition in *Aars2*‐knockout tumors compared with controls, with more visible localization of CD8+ T cells and NK cells in tumor tissues (Figure 2h). These spatial data suggest that *Aars2* loss may suppress tumor growth by reshaping the infiltration and localization of cytotoxic immune cells.

To test whether the antitumor effect caused by *Aars2* loss depends on CD8+ T cells and NK cells, we performed in vivo immune cell depletion experiments. EO771 control cells or *Aars2*‐knockout cells were first implanted subcutaneously into mice, followed by treatment with anti‐CD8 or anti‐NK1.1 antibodies to deplete CD8+ T cells or NK cells, respectively (Figure 2i). Flow cytometry confirmed that anti‐CD8 treatment efficiently reduced the proportion of CD8+ T cells (Figure 2j). However, after CD8+ T cell depletion alone, tumor volume and endpoint tumor weight in the *Aars2*‐deficient group remained lower than those in the control group (Figure 2k,l), indicating that depletion of CD8+ T cells alone could not fully reverse the antitumor effect of *Aars2* loss. Similarly, anti‐NK1.1 treatment efficiently depleted NK cells (Figure 2m), but NK cell depletion alone also did not fully reverse the suppression of tumor growth caused by *Aars2* loss (Figure 2n,o). Only when CD8+ T cells and NK cells were depleted together did the difference in tumor growth between control and *Aars2*‐deficient tumors largely disappear (Figure 2p,q). These results indicate that the in vivo antitumor effect of *Aars2* loss is not determined by a single immune cell population but depends on the combined cytotoxic pressure of CD8+ T cells and NK cells.

To test whether this mechanism is conserved in human tumors, we knocked out *AARS2* in MDA‐MB‐231 human breast cancer cells and implanted the cells into the mammary fat pads of immunodeficient NCG mice. Tumor‐specific CD8+ T cells, NK cells, or both cell types were then transferred through the tail vein (Figure 2r). In the absence of immune cell transfer, *AARS2* loss had little effect on MDA‐MB‐231 tumor growth or endpoint tumor weight (Figure 2s,t). However, after transfer of CD8+ T cells or NK cells, *AARS2* loss significantly strengthened immune cell‐mediated suppression of tumor growth. The largest reduction in tumor burden was observed when the two immune cell types were transferred together (Figure 2u–z). These data indicate that AARS2‐mediated immune escape is conserved between mouse and human tumor systems and that AARS2 is a key tumor cell‐intrinsic regulator that protects tumor cells from cooperative killing by CD8+ T cells and NK cells.

### 2.3 AARS2 Protects Tumor Cells From CD8+ T Cell and NK Cell Killing

To directly assess the effect of AARS2 on cytotoxic immune killing, we first measured tumor cell death in coculture systems containing human tumor cells and CD8+ T cells. After *AARS2* knockout in MDA‐MB‐231 breast cancer cells, tumor cell death was significantly higher than that in control cells at an effector‐to‐target ratio of 3:1 (Figure 3a). The same trend was observed in A375 melanoma cells, indicating that *AARS2* loss broadly increases the sensitivity of human tumor cells to CD8+ T cell killing (Figure 3b). Flow cytometry further showed that the proportions of granzyme B (GzmB)‐positive and interferon‐gamma (IFN‐γ)‐positive CD8+ T cells were markedly increased after coculture with *AARS2*‐deficient MDA‐MB‐231 cells, suggesting that *AARS2* loss enhances CD8+ T cell effector function (Figure 3c,d). In a mouse antigen‐specific system, *Aars2* loss also significantly increased tumor cell death after EO771‐OVA tumor cells were cocultured with OT‐I CD8+ T cells (Figure 3e). These cross‐species findings indicate that AARS2 contributes to tumor immune escape by negatively regulating tumor cell responses to CD8+ T cell cytotoxicity.

**FIGURE 3:**
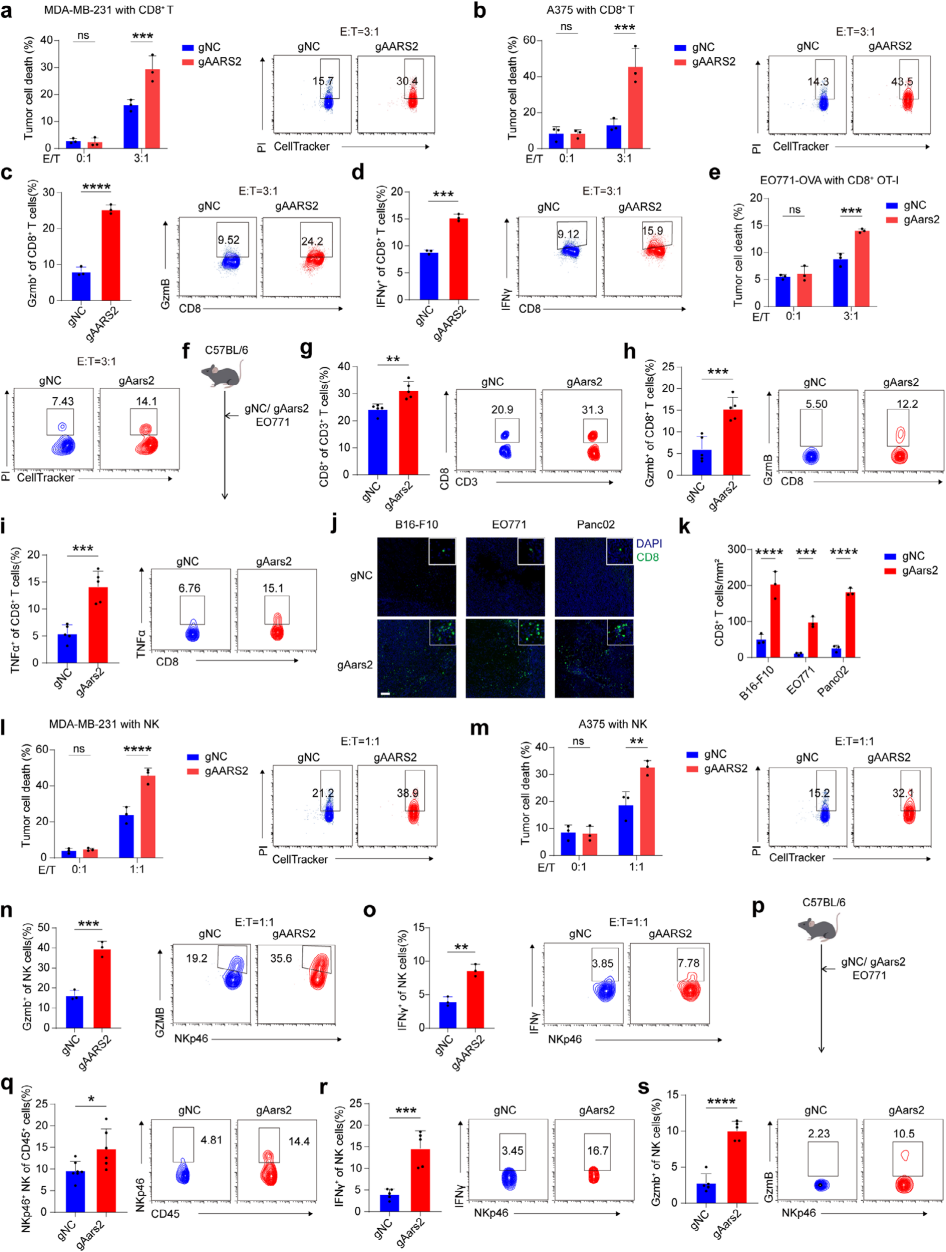
*AARS2* deficiency enhances tumor cell sensitivity to CD8+ T cell and NK cell cytotoxicity. (a, b) CD8+ T cell‐mediated cytotoxicity assays in control gNC or g*AARS2* MDA‐MB‐231 cells, ***p = 0.0009. (a) and A375 cells, ***p = 0.0003. (b). Left, quantification. Right, representative flow cytometry plots. (c, d) Flow cytometry analysis of CD8+ T cell effector function after co‐culture with control gNC or g*AARS2* MDA‐MB‐231 cells. Left, quantification of GzmB+ CD8+ T cells, ****p < 0.0001. (c) and IFN‐γ+ CD8+ T cells, ***p = 0.0001. (d). Right, representative flow cytometry plots. (e) Antigen‐specific CD8+ T cell‐mediated cytotoxicity assay using control gNC or g*Aars2* EO771‐OVA cells cocultured with OT‐I CD8+ T cells. Top, quantification. Bottom, representative flow cytometry plots. ***p = 0.0003. (f–i) Flow cytometry analysis of tumor‐infiltrating CD8+ T cells in C57BL/6 mice bearing control gNC or g*Aars2* EO771 tumors. f, Experimental scheme. g‐i, Left, quantification of CD8+ T cells among CD3+ T cells, **p = 0.0029. (g), GzmB+ cells among CD8+ T cells, ***p = 0.0008. (h) and TNF‐α+ cells among CD8+ T cells, ***p = 0.0004. (i). Right, representative flow cytometry plots. *n* = 5 mice per group. (j, k) Representative immunofluorescence images (j) and quantification (k) of CD8+ T cell infiltration in B16‐F10, EO771, and Panc02 tumors generated from control gNC or g*Aars2* cells. ***p = 0.0009, ****p < 0.0001. (l, m) NK cell‐mediated cytotoxicity assays in control gNC or g*AARS2* MDA‐MB‐231 cells, ****p < 0.0001. (l) and A375 cells, **p = 0.0023. (m). Left, quantification. Right, representative flow cytometry plots. (n, o) Flow cytometry analysis of NK cell effector function after co‐culture with control gNC or g*AARS2* tumor cells. Left, quantification of GzmB+ NK cells, ***p = 0.0009. (n) and IFN‐γ+ NK cells, **p = 0.0021. (o). Right, representative flow cytometry plots. (p–s) Flow cytometry analysis of tumor‐infiltrating NK cells in C57BL/6 mice bearing control gNC or g*Aars2* EO771 tumors. *p*, Experimental scheme. q‐s, Left, quantification of NKp46+ NK cells among CD45+ immune cells, *p = 0.0167. (q), IFN‐γ+ cells among NK cells, *** *p* = 0.0002. (r) and GzmB+ cells among NK cells, ****p < 0.0001. (s). Right, representative flow cytometry plots. *n* = 5 mice per group. Results are expressed as mean ± s.d. Each dot represents an independent biological replicate or mouse. P values were determined by two‐tailed unpaired t‐tests for two‐group comparisons and two‐way ANOVA with multiple‐comparisons test for effector‐to‐target ratio experiments. *p < 0.05, **p < 0.01, ***p < 0.001, ****p < 0.0001; ns, not significant.

We next tested the effect of Aars2 on CD8+ T cell infiltration and functional status in immunocompetent mice. Flow cytometry showed that the proportion of CD8+ T cells among CD3+ T cells was markedly increased in *Aars2*‐deficient EO771 tumors. The proportions of GzmB‐positive and tumor necrosis factor‐alpha (TNF‐α)‐positive infiltrating CD8+ T cells were also increased (Figure 3f–i). Immunofluorescence staining in B16‐F10, EO771, and Panc02 tumor models further showed that *Aars2* loss increased the density of CD8+ T cell infiltration in tumor tissues (Figure 3j,k). These in vivo results indicate that tumor cell‐derived AARS2 restricts CD8+ T cell entry into tumors and weakens their cytotoxic function.

We further examined the effect of AARS2 on NK cell‐mediated killing. In human tumor cell coculture systems, stable *AARS2* knockout in MDA‐MB‐231 breast cancer cells and A375 melanoma cells markedly increased cell death after 4 h of coculture with primary NK cells isolated and expanded from human peripheral blood mononuclear cells (Figure 3l,m). NK cells cocultured with *AARS2*‐deficient tumor cells also showed higher GzmB and IFN‐γ expression, indicating that *AARS2* loss enhances the NK cell effector state (Figure 3n,o). In the EO771 in vivo model, *Aars2*‐deficient tumors contained a higher proportion of NK cells, and these NK cells showed increased IFN‐γ and GzmB positivity (Figure 3p–s). Together, these data show that AARS2 helps tumor cells evade dual surveillance by adaptive and innate immunity by suppressing the infiltration and killing functions of both CD8+ T cells and NK cells.

### 2.4 AARS2 Promotes Tumor Immune Escape by Maintaining CD112 Stability

Cancer cells have been reported to evade CD8+ T cell and NK cell immune surveillance by expressing multiple immunosuppressive ligands [24, 25, 26, 27, 28]. To identify downstream molecules through which AARS2 affects CD8+ T cell and NK cell killing, we first performed proteomic analysis of *AARS2*‐deficient and control tumor cells (Figure 4a). Gene set enrichment analysis showed that immunosuppression‐related molecules were globally reduced in *AARS2*‐deficient cells, suggesting that AARS2 may regulate immune cell function by controlling immunosuppressive ligands on the tumor cell surface (Figure 4b,c). Based on the proteomic results and previously reported immune‐regulatory functions, we further screened multiple ligand molecules linked to CD8+ T cell or NK cell escape. The CD8+ T cell escape‐related molecules included the canonical immune checkpoint molecules B7‐H3, PD‐L1, and VISTA; the costimulatory ligand CD70; the lectins Galectin‐3 and Galectin‐9; and the adhesion molecules CD112 and CD155. The NK cell escape‐related inhibitory ligands included BAG‐6, CD112, CD155, CDH1, CLEC2D, HLA‐C, HLA‐E, NKTR, and SUMO2.

**FIGURE 4:**
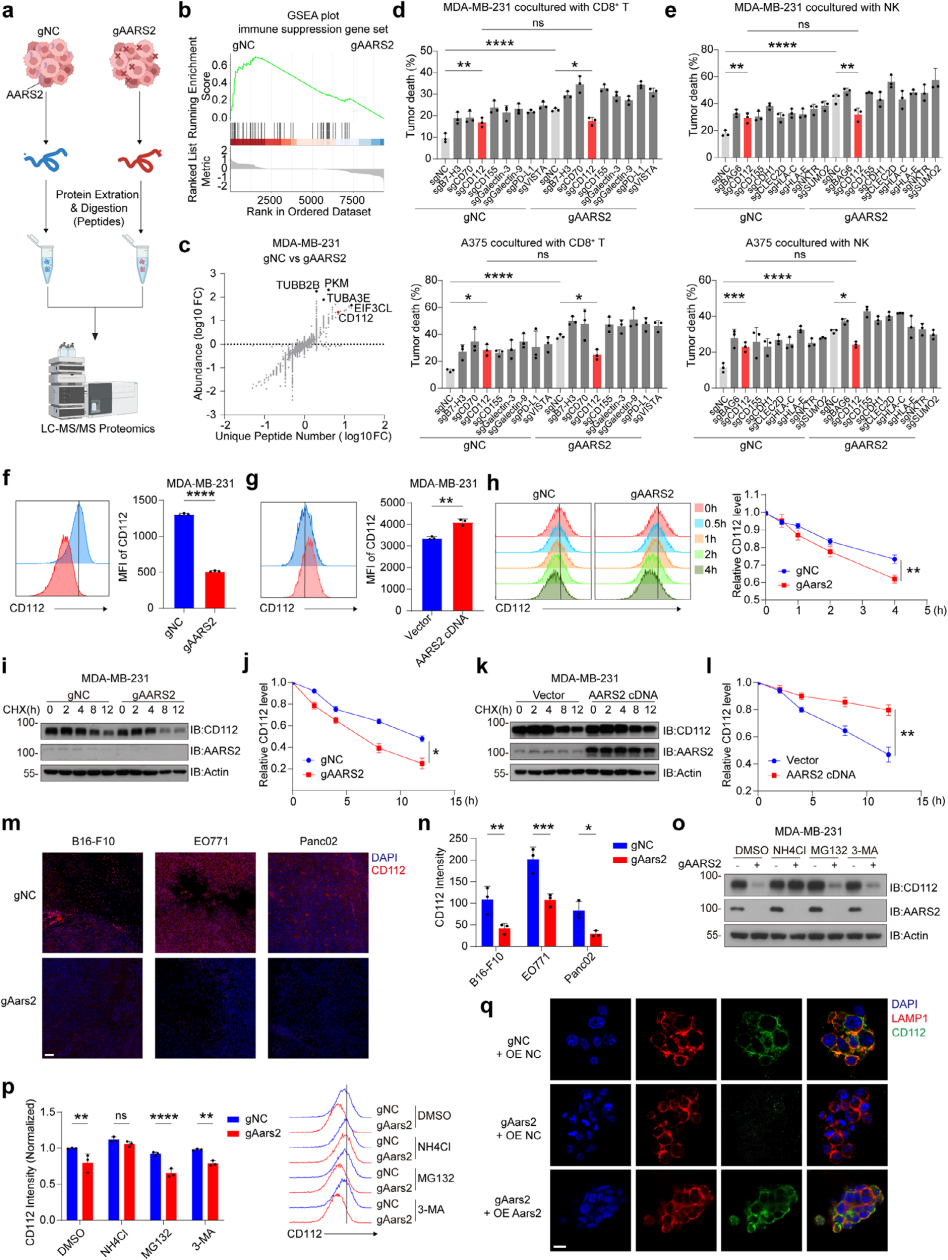
AARS2 maintains CD112 protein stability to mediate tumor immune escape. (a–c) Proteomics‐based screening of immune regulatory molecules downstream of AARS2. (a) Schematic of LC‐MS/MS proteomic analysis in control gNC and g*AARS2* tumor cells. (b) Gene set enrichment analysis of the immune suppression gene set in g*AARS2* versus gNC cells. (c) Proteomic analysis of proteins with altered abundance in MDA‐MB‐231 g*AARS2* versus gNC cells, with CD112 labeled as a candidate protein potentially affected by *AARS2* depletion. (d, e) Epistasis analysis of candidate immune regulatory ligands in CD8+ T cell‐mediated killing (d) and NK cell‐mediated killing (e) assays. MDA‐MB‐231 and A375 cells with control gNC or g*AARS2* background were further targeted with sgRNAs against the indicated immune regulatory genes and then cocultured with CD8+ T cells or NK cells. (f, g) Flow cytometry analysis of cell surface CD112 levels in MDA‐MB‐231 cells after *AARS2* knockout, ****p < 0.0001. (f) or *AARS2* cDNA expression, **p = 0.0013. (g). Left, representative flow cytometry histograms. Right, quantification of CD112 mean fluorescence intensity (MFI). (h) Cycloheximide (CHX) chase assay monitoring cell surface CD112 degradation in control gNC and g*AARS2* MDA‐MB‐231 cells. Left, representative flow cytometry histograms at the indicated time points. Right, quantification of relative CD112 levels. **p = 0.0093. (i, j) CHX chase assay by Western blot showing total CD112 protein degradation in control gNC and g*AARS2* MDA‐MB‐231 cells. (i) Representative Western blots. (j) Quantification of relative CD112 levels, *p = 0.0208. (k, l) CHX chase assay by Western blot showing total CD112 protein degradation in MDA‐MB‐231 cells expressing empty vector or *AARS2* cDNA. (k) Representative Western blots. (l) Quantification of relative CD112 levels. **p = 0.0078. (m, n) Representative immunofluorescence images (m) and quantification (n) of CD112 expression in B16‐F10, EO771, and Panc02 tumors generated from control gNC or g*Aars2* cells. Scale bar, 50 µm. *p = 0.0251, **p = 0.0067, ***p = 0.0004. (o) Western blot analysis of CD112 protein levels in control gNC and g*AARS2* MDA‐MB‐231 cells treated with DMSO, the lysosomal inhibitor NH4Cl, the proteasome inhibitor MG132, or the autophagy inhibitor 3‐MA. (p) Flow cytometry analysis of cell surface CD112 levels in control gNC and g*AARS2* MDA‐MB‐231 cells after the indicated inhibitor treatments. Left, quantification. Right, representative flow cytometry histograms. **p = 0.0014; ****p < 0.0001; **p = 0.0021. (q) Immunofluorescence imaging showing redistribution of CD112 from the plasma membrane to late endosomes/lysosomes (LAMP1+ compartments) upon *AARS2* knockout. Scale bar, 10 µm. Results are expressed as mean ± s.d. Each dot represents an independent biological replicate. P values were determined by two‐tailed unpaired t‐tests for two‐group comparisons, one‐way ANOVA with multiple‐comparisons test for multi‐group comparisons, and two‐way ANOVA with multiple‐comparisons test for CHX chase assays. *p < 0.05, **p < 0.01, ***p < 0.001, ****p < 0.0001; ns, not significant. Original blot images corresponding to the immunoblotting panels in this figure are provided in Figure S1.

To identify immune‐regulatory ligands that mediate *AARS2*‐dependent immune resistance, we individually disrupted candidate ligands in control and *AARS2*‐knockout MDA‐MB‐231 and A375 cells. Among the tested CD8+ T cell‐related molecules, *CD112* depletion increased tumor cell sensitivity to CD8+ T cell killing. Importantly, combined knockout of *AARS2* and *CD112* did not further enhance CD8+ T cell‐mediated killing compared with *CD112* knockout alone, indicating that CD112 is required for the immune‐sensitizing effect caused by *AARS2* loss (Figure 4d). Similarly, parallel screening of NK cell‐related inhibitory ligands identified CD112 as a key molecule required for *AARS2*‐dependent resistance to NK cell killing, because *AARS2* loss failed to provide additional sensitization in *CD112*‐deficient tumor cells (Figure 4e). Proteomic analysis also confirmed that *AARS2* knockout caused a significant reduction in CD112 protein levels in cancer cells (Figure 4c).

CD112, also known as NECTIN‐2 or PVRL2, is an immunoglobulin‐like transmembrane adhesion molecule. Tumor cells with high CD112 expression preferentially bind the inhibitory receptors CD112R and TIGIT on immune cells, inhibit T and NK cell activation through SHP‐1/2‐mediated dephosphorylation, block IFN‐γ production, and establish an “immune brake” [29, 30, 31, 32]. We next directly measured the effect of AARS2 on CD112 protein expression. Flow cytometry showed that *AARS2* loss significantly reduced CD112 levels on the surface of MDA‐MB‐231 cells, whereas *AARS2* overexpression increased CD112 expression (Figure 4f,g). To define how AARS2 regulates CD112 protein levels, we used cycloheximide (CHX) to block new protein synthesis and then tracked CD112 degradation. *AARS2* loss accelerated the decline of membrane CD112 (Figure 4h) and total CD112 protein (Figure 4i,j), whereas *AARS2* overexpression delayed CD112 degradation (Figure 4k,l). Immunofluorescence staining of B16‐F10, EO771, and Panc02 mouse tumors further confirmed that *Aars2* loss reduced CD112 protein signals (Figure 4m,n). These findings indicate that AARS2 maintains CD112 expression mainly by increasing CD112 protein stability.

To further define the degradation pathway of CD112, we treated cells with the lysosomal acidification inhibitor NH4Cl, the proteasome inhibitor MG132, or the autophagy inhibitor 3‐MA. Only lysosome blockade clearly restored CD112 protein levels in *AARS2*‐deficient cells, indicating that CD112 is mainly degraded through the lysosomal pathway (Figure 4o). Flow cytometric measurement of cell‐surface CD112 showed the same result: only lysosome blockade clearly restored CD112 protein levels in *AARS2*‐deficient cells (Figure 4p). To further verify that AARS2 controls lysosomal degradation of CD112, immunofluorescence tracing showed that CD112 was mainly localized on the plasma membrane in control cells. After *AARS2* loss, more CD112 entered LAMP1‐positive late endosome/lysosome structures, whereas *AARS2* re‐expression restored CD112 membrane localization (Figure 4q). Thus, AARS2 stabilizes membrane CD112 by limiting its lysosomal degradation, providing a molecular basis for tumor escape from CD8+ T cell and NK cell killing.

### 2.5 AARS2 Drives Tumor Immune Escape by Mediating CD112 Lactylation

AARS2, an aminoacyl‐tRNA synthetase involved in protein translation, has recently been shown to function as both a lactate sensor and a lactyltransferase [19, 33]. To test whether AARS2 regulates immune escape through lactate‐dependent lactylation, we first altered intracellular lactate metabolism in tumor cells. Intracellular L‐lactate is mainly generated from pyruvate by lactate dehydrogenase A (LDHA) [20, 34], whereas lactate transport across the plasma membrane is mediated by monocarboxylate transporters, including MCT1 and MCT4 [35]. Reducing lactate production with the glycolysis inhibitor 2‐DG or the lactate dehydrogenase inhibitor oxamate made MDA‐MB‐231 cells more sensitive to CD8+ T cell killing (Figure 5a). In contrast, blocking lactate export with the MCT1 inhibitors ARC155858 (ARC) or AZD3965 (AZD), which causes intracellular lactate accumulation, reduced CD8+ T cell‐mediated tumor cell death (Figure 5b). Notably, in *AARS2*‐deficient cells, these lactate metabolism interventions no longer significantly changed CD8+ T cell killing, indicating that the effect of lactate on T cell killing sensitivity depends on AARS2 (Figure 5c,d).

**FIGURE 5:**
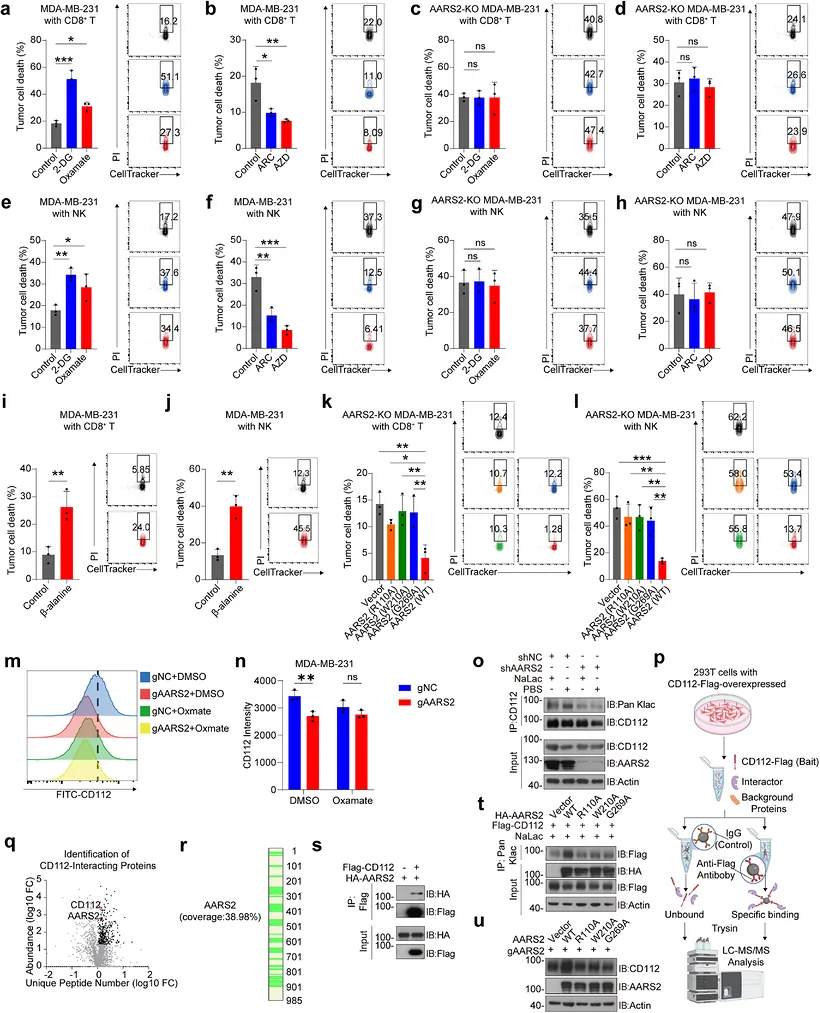
AARS2‐mediated Lactylation of CD112 promotes tumor immune evasion. (a, b) CD8+ T cell‐mediated killing of MDA‐MB‐231 cells after lactate metabolic modulation. Tumor cells were treated with 2‐DG or oxamate to reduce lactate production, *p = 0.0181, ***p = 0.0001. (a) or with the MCT1 inhibitors ARC155858 (ARC) or AZD3965 (AZD) to increase intracellular lactate accumulation, *p = 0.0165, **p = 0.0055. (b). Left, quantification. Right, representative flow cytometry plots. (c, d) CD8+ T cell‐mediated killing of *AARS2*‐knockout MDA‐MB‐231 cells after treatment with 2‐DG or oxamate (c) or with ARC or AZD (d). Left, quantification. Right, representative flow cytometry plots. (e, f) NK cell‐mediated killing of MDA‐MB‐231 cells after treatment with 2‐DG or oxamate, *p = 0.0377, **p = 0.0057. (e) or with ARC or AZD, **p = 0.0028, ***p = 0.0005. (f). Left, quantification. Right, representative flow cytometry plots. (g, h) NK cell‐mediated killing of *AARS2*‐knockout MDA‐MB‐231 cells after treatment with 2‐DG or oxamate (g) or with ARC or AZD (h). Left, quantification. Right, representative flow cytometry plots. (i, j) Tumor cell killing after competitive inhibition of AARS2 lactate binding by β‐alanine in CD8+ T cell coculture, **p = 0.0097. (i) and NK cell co‐culture, **p = 0.0026. (j) assays. Left, quantification. Right, representative flow cytometry plots. (k, l) Rescue analysis of AARS2‐dependent immune resistance in *AARS2*‐knockout MDA‐MB‐231 cells reconstituted with empty vector, wild‐type *AARS2*, or lactyltransferase‐defective *AARS2* mutants. Tumor cell death was measured after coculture with CD8+ T cells, Vector vs. WT, **p = 0.0016; AARS2 (R110A) vs. WT, *p = 0.0321; AARS2 (W210A) vs. WT, **p = 0.0044; AARS2 (G269A) vs. WT, **p = 0.0053. (k) or NK cells, Vector vs. WT, ***p = 0.0006; AARS2 (R110A) vs. WT, **p = 0.0026; AARS2 (W210A) vs. WT, **p = 0.0027; AARS2 (G269A) vs. WT, **p = 0.0049. (l). Left, quantification. Right, representative flow cytometry plots. (m, n) Flow cytometry analysis of cell surface CD112 levels in control gNC and g*Aars2* MDA‐MB‐231 cells after DMSO or oxamate treatment. m, Representative flow cytometry histograms. n, Quantification of CD112 mean fluorescence intensity. **p = 0.0037. (o) Immunoprecipitation analysis of CD112 lactylation in MDA‐MB‐231 cells after NaLac treatment and *AARS2* knockdown. CD112 was immunoprecipitated and probed with pan‐lysine lactylation antibody. (p–r) Identification of CD112‐interacting proteins by immunoaffinity purification and LC‐MS/MS. p, Schematic of CD112‐Flag pull‐down and LC‐MS/MS analysis in CD112‐overexpressing 293T cells. q, Proteomic plot of CD112‐interacting proteins, with AARS2 labeled as a candidate CD112‐associated protein. (r) Peptide sequence coverage of AARS2 identified by LC‐MS/MS. (s) Co‐immunoprecipitation assay confirming the interaction between Flag‐CD112 and HA‐AARS2 in 293T cells. (t) Immunoprecipitation analysis of CD112 lactylation in 293T cells expressing Flag‐CD112 together with empty vector, wild‐type *AARS2* or lactyltransferase‐defective *AARS2* mutants under NaLac treatment. (u) Western blot analysis of CD112 protein levels in *AARS2*‐knockout MDA‐MB‐231 cells reconstituted with empty vector, wild‐type *AARS2* or lactyltransferase‐defective *AARS2* mutants. Results are expressed as mean ± s.d. Each dot represents an independent biological replicate. P values were determined by two‐tailed unpaired t‐tests for two‐group comparisons, one‐way ANOVA with multiple‐comparisons test for multi‐group comparisons, and two‐way ANOVA with multiple‐comparisons test where applicable. *p < 0.05, **p < 0.01, ***p < 0.001; ns, not significant. Original blot images corresponding to the immunoblotting panels in this figure are provided in Figure S1.

Similar results were observed in NK cell co‐culture systems. Reducing lactate production enhanced NK cell‐mediated tumor cell death, whereas intracellular lactate accumulation induced by MCT1 inhibitors reduced NK cell killing efficiency. These effects largely disappeared in the *AARS2*‐deficient background (Figure 5e–h). In addition, β‐alanine, a competitive inhibitor of lactate binding to AARS1/2 20, enhanced both CD8+ T cell and NK cell killing of tumor cells (Figure 5i,j). These findings show that AARS2‐mediated tumor immune escape is closely linked to intracellular lactate levels.

To determine whether the lactyltransferase activity of AARS2 is required for immune escape, we reconstituted *AARS2*‐deficient cells with wild‐type AARS2 or lactyltransferase‐defective mutants (R110A, W210A, and G269A), which selectively abolish the lactylation activity of AARS2 [19]. Re‐expression of wild‐type *AARS2* in *AARS2*‐deficient tumor cells restored resistance to CD8+ T cell and NK cell killing, whereas none of the three activity‐defective mutants restored this phenotype (Figure 5k,l). These results indicate that AARS2 helps tumor cells evade immune surveillance through its lactyltransferase activity and the lactylation of downstream target proteins.

Because CD112 is a key downstream molecule of AARS2, we next tested whether AARS2 regulates CD112 stability through lactylation. In tumor cells, blocking endogenous lactate production with oxamate significantly reduced CD112 protein levels, whereas CD112 changed only slightly in *AARS2*‐deficient cells (Figure 5m,n). In MDA‐MB‐231 cells, CD112 lactylation was detectable under basal culture conditions. Exogenous NaLac treatment produced only a modest, statistically nonsignificant change in CD112 lactylation. In contrast, AARS2 knockdown reduced CD112 lactylation under both basal and NaLac‐treated conditions, supporting a role for AARS2 in mediating CD112 lactylation (Figure 5o). To identify potential regulators of CD112 lactylation, we performed immunoaffinity purification followed by LC‐MS/MS in 293T cells overexpressing *CD112*. AARS2 was identified as the major CD112‐interacting protein, whereas previously reported lactylation‐related enzymes, including lactyl‐CoA synthetases such as ACSS2 and GTPSCS [18, 36, 37, 38, 39, 40], and KAT‐family lactyltransferases such as p300, CBP, KAT2A, TIP60/KAT5, HBO1/KAT7, and KAT8 [38, 41], were not detected as CD112‐interacting proteins (Figure 5p–r). Co‐immunoprecipitation further validated the interaction between CD112 and AARS2 (Figure 5s). To determine whether AARS2 mediates CD112 lactylation, we performed overexpression experiments in 293T cells. Wild‐type AARS2 markedly increased CD112 lactylation under lactate stimulation, whereas the lactyltransferase‐defective mutants R110A, W210A, and G269A failed to efficiently induce CD112 lactylation (Figure 5t). To determine whether AARS2 lactyltransferase activity is required to maintain CD112 protein abundance in tumor cells, we reconstituted *AARS2*‐deficient MDA‐MB‐231 cells with wild‐type *AARS2* or lactyltransferase‐defective mutants. Re‐expression of wild‐type AARS2 restored CD112 protein levels, whereas R110A, W210A, or G269A mutants failed to do so (Figure 5u). Together, these results indicate that AARS2 promotes lactate‐dependent CD112 lactylation and that its lactyltransferase activity is required for maintaining CD112 protein stability.

Previous studies showed that AARS2 can bind and lactylate cGAS under lactate stimulation, thereby suppressing the cGAS‐STING pathway [19]. To test whether AARS2 affects antitumor immunity through this pathway, we examined related signaling molecules after *Aars2* loss and lactate metabolism interventions. HT‐DNA stimulation clearly induced phosphorylation of IRF3, STAT1, and TBK1, indicating that the cGAS‐STING‐IFN pathway was responsive in this system. However, under β‐alanine, 2‐DG, or ARC treatment, *Aars2* loss did not consistently enhance phosphorylation of these molecules (Figure S2a–c). RNA‐seq, qPCR, and proteomic analyses further showed that *Cgas*, *Tmem173*, *Stat1*, and multiple interferon‐stimulated genes were not consistently upregulated after *Aars2* loss. The mRNA levels of *Isg56*, *Cxcl10*, and *Ifnb* also showed no significant differences (Figure S2d–f). These data indicate that AARS2 loss does not markedly activate the cGAS–STING–IFN pathway in the models tested here. Together with our genetic and biochemical findings, these results support CD112 as a functionally validated downstream effector of AARS2‐mediated immune evasion in this context, while not excluding contributions from other AARS2 substrates in different cellular settings.

### 2.6 AARS2‐Mediated CD112 Lactylation Disrupts Its Interaction With UBE3C

Because multiple lysine post‐translational modifications regulate protein stability by altering ubiquitination [42, 43], we first examined whether CD112 lactylation affects CD112 ubiquitination. In 293T cells coexpressing CD112‐FLAG and HA‐Ub, sodium lactate treatment induced a moderate reduction in CD112 ubiquitination (Figure 6a). Because this experiment was performed in an overexpression system, in which the high abundance of ectopically expressed CD112 may attenuate the apparent effect of lactylation on ubiquitination, we next evaluated this relationship under endogenous conditions in tumor cells by modulating lactate metabolism. Treatment with oxamate or β‐alanine reduced endogenous CD112 lactylation (Figure 6b), increased CD112 ubiquitination (Figure 6c), and decreased CD112 protein stability and abundance (Figure 6d). Conversely, inhibition of lactate export with the MCT1 inhibitors ARC (AR‐C155858) or AZD (AZD3965) promoted intracellular lactate accumulation, enhanced endogenous CD112 lactylation (Figure 6e), suppressed CD112 ubiquitination (Figure 6f), and stabilized CD112 protein accumulation (Figure 6g). Together, these reciprocal changes under endogenous conditions support the physiological relevance of lactylation‐mediated suppression of CD112 ubiquitination.

**FIGURE 6:**
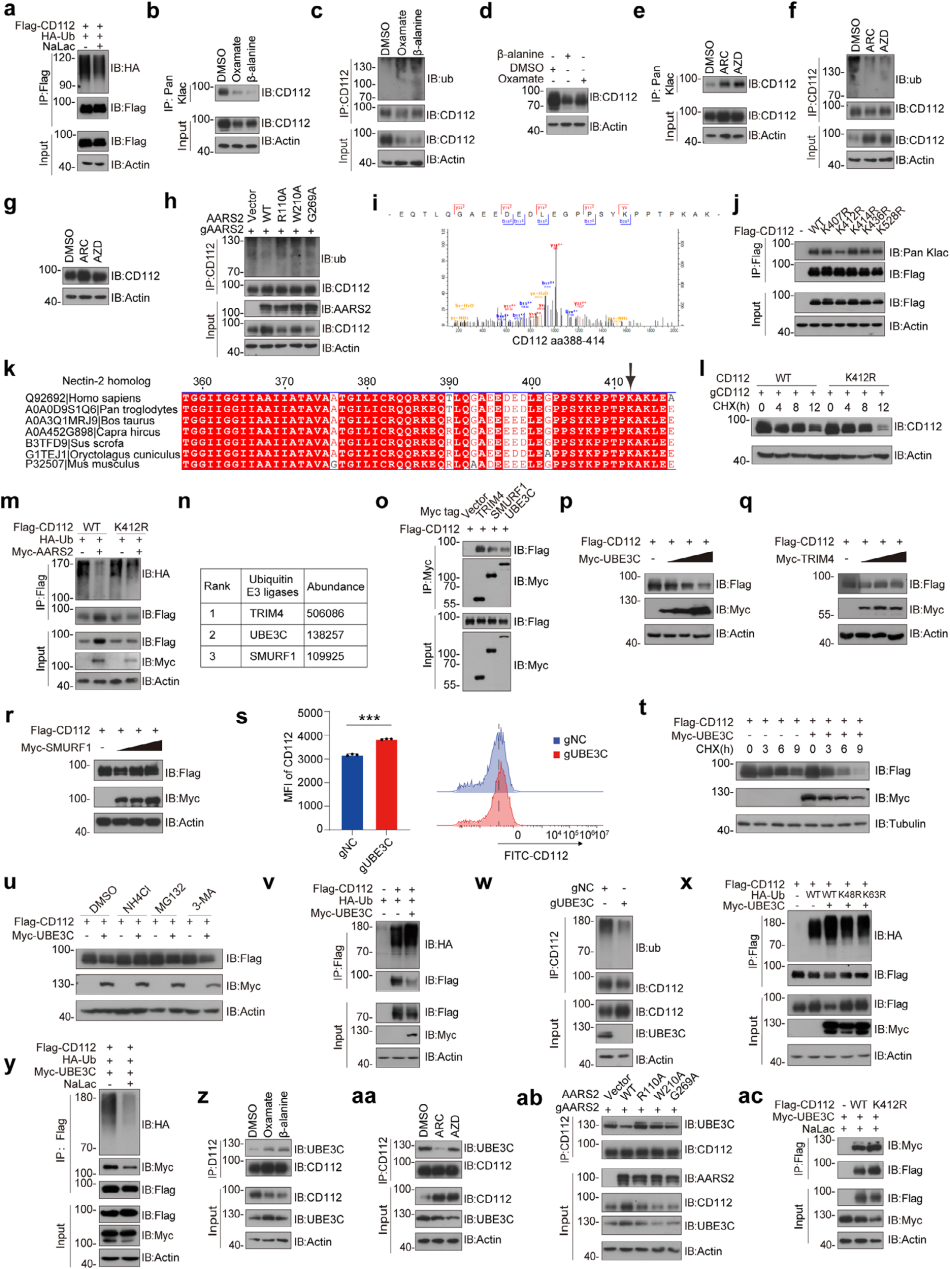
AARS2‐mediated CD112 lactylation blocks UBE3C‐dependent ubiquitination and maintains CD112 protein stability. (a) Immunoprecipitation analysis of CD112 ubiquitination in 293T cells expressing Flag‐CD112 and HA‐ubiquitin after sodium lactate (NaLac) treatment. (b–d) Effects of reduced intracellular lactate signaling on CD112 lactylation, ubiquitination, and protein abundance in MDA‐MB‐231 cells. b, Immunoprecipitation analysis of CD112 lactylation after oxamate or β‐alanine treatment. (c) Immunoprecipitation analysis of CD112 ubiquitination after oxamate or β‐alanine treatment. (d) Western blot analysis of CD112 protein levels after oxamate or β‐alanine treatment. (e–g) Effects of intracellular lactate accumulation on CD112 lactylation, ubiquitination, and protein abundance in MDA‐MB‐231 cells. Cells were treated with the MCT1 inhibitors ARC155858 (ARC) or AZD3965 (AZD). (e) Immunoprecipitation analysis of CD112 lactylation. (f) Immunoprecipitation analysis of CD112 ubiquitination. (g) Western blot analysis of CD112 protein levels. (h) Immunoprecipitation analysis of CD112 ubiquitination in *AARS2*‐knockout MDA‐MB‐231 cells reconstituted with empty vector, wild‐type *AARS2*, or lactyltransferase‐defective *AARS2* mutants. (i) LC‐MS/MS spectrum identifying CD112 K412 as a lactylation site. (j) Immunoprecipitation analysis of CD112 lactylation in cells expressing wild‐type CD112 or lysine‐to‐arginine CD112 mutants, identifying K412 as the main lactylation site. (k) Sequence alignment showing conservation of the CD112 K412 region across species. (l) Cycloheximide (CHX) chase assay comparing the stability of wild‐type CD112 and the CD112 K412R mutant. (m) Immunoprecipitation analysis of CD112 ubiquitination in cells expressing wild‐type CD112 or the CD112 K412R mutant with or without AARS2 expression. (n, o) Identification and validation of candidate E3 ubiquitin ligases interacting with CD112. (n) LC‐MS/MS‐based ranking of CD112‐associated E3 ligases. (o) Co‐immunoprecipitation assay testing the interaction between CD112 and candidate E3 ligases. (p–r) Western blot analysis of CD112 protein levels after increasing expression of UBE3C (p), TRIM4 (q), or SMURF1 (r). (s) Flow cytometry analysis of cell surface CD112 levels in control and UBE3C‐knockout MDA‐MB‐231 cells. Left, quantification of CD112 mean fluorescence intensity (MFI); right, representative flow cytometry histograms are shown. (t) CHX chase assay showing the effect of UBE3C on CD112 protein degradation. (u) Western blot analysis of CD112 protein levels in cells expressing UBE3C after treatment with DMSO, the lysosomal inhibitor NH4Cl, the proteasome inhibitor MG132, or the autophagy inhibitor 3‐MA. (v) Immunoprecipitation analysis of UBE3C‐mediated CD112 ubiquitination in 293T cells expressing Flag‐CD112, HA‐ubiquitin, and Myc‐UBE3C. (w) Immunoprecipitation analysis of endogenous CD112 ubiquitination in control and UBE3C‐knockout MDA‐MB‐231 cells. (x) Immunoprecipitation analysis of UBE3C‐mediated CD112 ubiquitination using wild‐type ubiquitin or ubiquitin mutants defective in K48‐ or K63‐linked chain formation. (y) Immunoprecipitation analysis showing the effect of NaLac treatment on CD112 ubiquitination and CD112‐UBE3C interaction in 293T cells. (z) (aa) Co‐immunoprecipitation analysis of endogenous CD112‐UBE3C interaction after lactate pathway modulation. (z) CD112‐UBE3C interaction after DMSO, oxamate, or β‐alanine treatment. aa, CD112‐UBE3C interaction after DMSO, ARC, or AZD treatment. (ab) Co‐immunoprecipitation analysis of CD112‐UBE3C interaction in *AARS2*‐knockout MDA‐MB‐231 cells reconstituted with empty vector, wild‐type *AARS2*, or lactyltransferase‐defective *AARS2* mutants. (ac) Co‐immunoprecipitation analysis comparing UBE3C binding to wild‐type CD112 and the CD112 K412R mutant under NaLac treatment. Data are presented as mean ± s.d. Each dot represents an independent biological replicate. *p*‐values were determined by a two‐tailed unpaired *t*‐test. *p < 0.05, **p < 0.01, ***p < 0.001, ****p < 0.0001; ns, not significant. IB, immunoblot; IP, immunoprecipitation; CHX, cycloheximide; NaLac, sodium lactate; ARC, ARC155858; AZD, AZD3965; Klac, lysine lactylation; Ub, ubiquitin. Original blot images corresponding to the immunoblotting panels in this figure are provided in Figure S1.

To define the role of AARS2 in this process, we first performed genetic rescue experiments. *AARS2* knockout in MDA‐MB‐231 cells significantly increased CD112 ubiquitination. Re‐expression of wild‐type *AARS2* suppressed CD112 ubiquitination, whereas overexpression of lactyltransferase‐defective mutants (R110A, W210A, or G269A) did not have this effect (Figure 6h). These results indicate that the lactyltransferase activity of AARS2 is required to suppress CD112 ubiquitination.

To further validate this conclusion, we used pharmacological intervention. Treatment of tumor cells with β‐alanine, a competitive antagonist that blocks lactate binding to AARS1/2, significantly reduced CD112 lactylation (Figure 6b), increased CD112 ubiquitination (Figure 6c), and accelerated CD112 protein degradation (Figure 6d). These genetic and pharmacological data support each other and confirm that AARS2 catalyzes CD112 lactylation through its lactyltransferase activity, thereby opposing ubiquitination.

To determine how AARS2‐dependent lactylation suppresses CD112 ubiquitination, we identified the lactylation sites of CD112 by mass spectrometry. CD112 was lactylated at K412 in MDA‐MB‐231 cells (Figure 6i). Because AARS2 is mainly localized intracellularly, we mutated five lysine residues in the intracellular region of CD112 (K407, K412, K414, K436, and K528) to arginine. The K412R mutation markedly reduced CD112 lactylation, confirming K412 as the major lactylation site (Figure 6j). Sequence alignment of CD112/NECTIN‐2 homologs from multiple mammalian species showed that K412 and its surrounding cytoplasmic residues are highly conserved, supporting the potential functional importance of this lactylation site (Figure 6k).

To define how CD112 K412 lactylation affects protein stability, we measured the degradation rate and ubiquitination level of the K412R mutant. The K412R mutation markedly reduced CD112 expression, shortened its half‐life, and increased its ubiquitination (Figure 6l,m). These results indicate that CD112 K412 is the central site regulated by AARS2‐mediated lactylation and that the lactylation status of this site directly determines CD112 protein stability.

### 2.7 CD112 Lactylation Blocks Ubiquitin‐Dependent Degradation by Weakening UBE3C Binding

To clarify the molecular mechanism by which lactylation and ubiquitination compete to control CD112 protein levels, we first identified the E3 ubiquitin ligase responsible for CD112 ubiquitination by mass spectrometry of CD112‐interacting proteins. TRIM4, UBE3C, and SMURF1 emerged as candidate E3 ligases with strong CD112 binding (Figure 6n). Co‐immunoprecipitation confirmed that all three candidate proteins interacted with CD112 (Figure 6o). Because both mass spectrometry and co‐immunoprecipitation suggested that TRIM4, UBE3C, and SMURF1 could bind CD112, we next compared their effects on CD112 protein expression. Only UBE3C overexpression markedly reduced CD112 protein levels in a dose‐dependent manner, whereas TRIM4 and SMURF1 overexpression did not cause a clear decrease in CD112. These results indicate that among the candidate E3 ubiquitin ligases, UBE3C is the functional E3 ligase mainly responsible for promoting CD112 protein degradation (Figure 6p–r). Flow cytometry further showed that UBE3C loss significantly increased cell‐surface CD112 expression in MDA‐MB‐231 cells (Figure 6s). CHX chase experiments showed that UBE3C overexpression accelerated CD112 protein loss and shortened its half‐life (Figure 6t). Therefore, UBE3C was selected for further analysis as the main candidate E3 ubiquitin ligase that mediates CD112 ubiquitination and degradation.

To explore the pathway through which UBE3C degrades CD112, we treated cells with NH4Cl, MG132, or 3‐MA. NH4Cl clearly rescued the UBE3C overexpression‐induced decrease in CD112, further supporting that UBE3C promotes CD112 degradation through a lysosome‐associated pathway (Figure 6u). We then directly measured the effect of UBE3C on CD112 ubiquitination. In 293T cells coexpressing CD112, HA‐Ub, and UBE3C, CD112 ubiquitination was markedly increased (Figure 6v). At the endogenous level, UBE3C knockout reduced CD112 ubiquitination and was accompanied by increased CD112 protein levels (Figure 6w). Because HECT‐family E3 ligases can determine ubiquitin‐chain linkage specificity and may generate distinct polyubiquitin chain types [42, 43], we used ubiquitin mutants and found that either the K48R or K63R mutation weakened UBE3C‐mediated CD112 ubiquitination (Figure 6x). These results indicate that UBE3C promotes K48‐ and K63‐linked polyubiquitination of CD112, thereby driving CD112 lysosomal degradation and negatively regulating CD112 protein levels.

To determine how AARS2‐mediated CD112 lactylation affects UBE3C‐dependent ubiquitination, we co‐expressed HA‐Ub, CD112‐FLAG, and UBE3C‐Myc in 293T cells. Sodium lactate treatment significantly suppressed CD112 ubiquitination and markedly weakened the interaction between CD112 and UBE3C (Figure 6y). Consistently, oxamate, which reduces lactate production, and β‐alanine, which blocks lactate–AARS binding, both increased the interaction between CD112 and UBE3C (Figure 6z), whereas the MCT1 inhibitors ARC and AZD both reduced this interaction, with ARC producing a greater reduction than AZD under the experimental conditions used (Figure 6aa). These results indicate that lactate‐associated CD112 lactylation is negatively associated with CD112–UBE3C binding.

Further mechanistic experiments showed that *AARS2* loss in MDA‐MB‐231 cells markedly enhanced the interaction between CD112 and UBE3C. Re‐expression of wild‐type *AARS2* weakened this interaction, whereas the lactyltransferase‐defective mutants R110A, W210A, and G269A did not have the same effect (Figure 6ab). Site‐specific experiments showed that the K412R mutation significantly enhanced CD112 binding to UBE3C (Figure 6ac).

Together, these findings show that AARS2‐mediated lactylation of CD112 at K412 creates a competitive molecular switch. When K412 is occupied by lactylation, UBE3C recognition of CD112 is blocked, which suppresses CD112 ubiquitination and lysosomal degradation and stabilizes CD112 protein. When lactylation is absent, UBE3C can bind CD112, catalyze its polyubiquitination, and promote its degradation.

### 2.8 AARS2‐High Tumors Respond to TIGIT×PD‐L1 Blockade and HSV‐2 Virotherapy

AARS2 stabilizes CD112 through lactylation, and CD112 is a functional ligand of the inhibitory receptor TIGIT. High CD112 expression can strengthen TIGIT‐mediated immunosuppressive signaling, inhibit the activation and killing functions of CD8+ T cells and NK cells, and promote tumor immune escape [29, 44, 45]. Therefore, in tumors with high *AARS2* expression, TIGIT blockade may release this inhibitory pathway and restore antitumor immune responses. Because TIGIT and PD‐1 are often coexpressed and generate cooperative inhibitory signaling, combined targeting of TIGIT and PD‐L1 may help overcome resistance to single‐agent therapy and provide a better immunotherapeutic strategy for *AARS2*‐high tumors [46, 47]. To combine the advantages of both antibodies, we constructed a TIGIT×PD‐L1 bispecific antibody (TIGIT×PD‐L1 BsAb) and used a knobs‐into‐holes Fc design to reduce the risk of heavy‐chain mismatch (Figure 7a).

**FIGURE 7:**
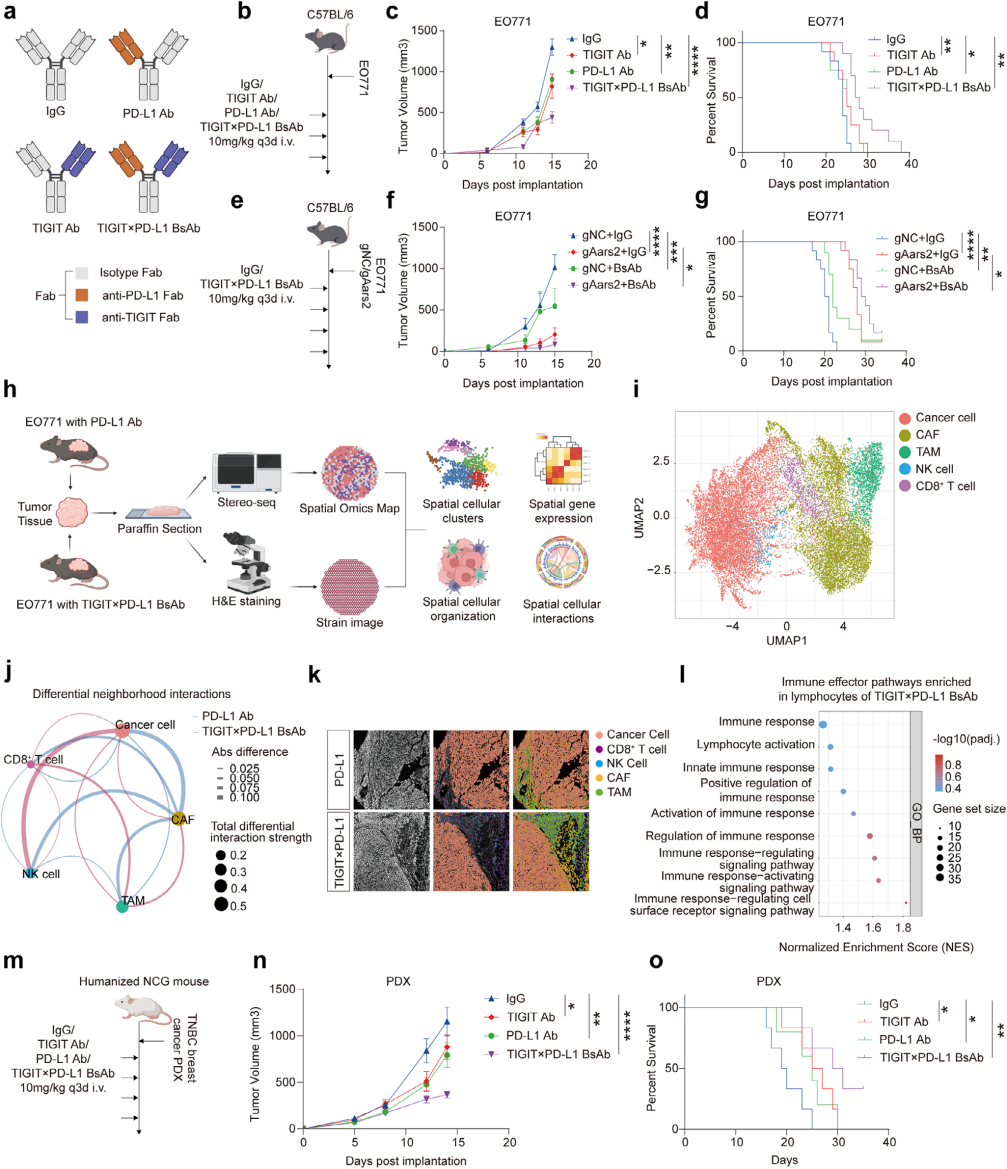
AARS2‐high tumors are sensitive to TIGIT×PD‐L1 bispecific antibody. (a) Schematic design of IgG, TIGIT antibody, PD‐L1 antibody, and TIGIT×PD‐L1 bispecific antibody (BsAb). (b–d) Therapeutic effects of IgG, TIGIT antibody, PD‐L1 antibody, or TIGIT×PD‐L1 BsAb in C57BL/6 mice bearing EO771 tumors. (b) Treatment scheme. (c) Tumor growth curves. IgG vs. TIGIT antibody, *p = 0.0113; IgG vs. PD‐L1 antibody, **p = 0.0097; IgG vs. TIGIT×PD‐L1 BsAb, ****p < 0.0001. (d) Kaplan–Meier survival curves. IgG vs. TIGIT antibody, **p = 0.0094; IgG vs. PD‐L1 antibody, *p = 0.01; IgG vs. TIGIT×PD‐L1 BsAb, **p = 0.0018. For c and d, *n* = 6–14 mice per group. (e–g) Therapeutic effects of TIGIT×PD‐L1 BsAb in C57BL/6 mice bearing control gNC or g*Aars2* EO771 tumors. (e) Treatment scheme. (f) Tumor growth curves. *p = 0.0252, ***p = 0.0006, ****p < 0.0001. (g) Kaplan–Meier survival curves. *p = 0.0227, **p = 0.0065, ****p < 0.0001. For f and g, *n* = 6–14 mice per group. (h–l) Spatial transcriptomic analysis of EO771 tumors treated with PD‐L1 antibody or TIGIT×PD‐L1 BsAb. (h) Workflow for Stereo‐seq spatial transcriptomics, H&E staining, and spatial cell–cell interaction analysis. (i) UMAP plot showing major cell populations, including cancer cells, CAFs, tumor‐associated macrophages (TAM), NK cells, and CD8+ T cells. (j) Differential neighborhood interaction analysis comparing PD‐L1 antibody and TIGIT×PD‐L1 BsAb treatment groups. (k) Representative spatial images showing the organization of cancer cells, CD8+ T cells, NK cells, CAFs, and TAMs in tumors treated with PD‐L1 antibody or TIGIT×PD‐L1 BsAb. (l) Gene set enrichment analysis showing immune effector pathways enriched in lymphocytes from TIGIT×PD‐L1 BsAb‐treated tumors. (m–o) Therapeutic effects of IgG, TIGIT antibody, PD‐L1 antibody, or TIGIT×PD‐L1 BsAb in a humanized NCG mouse model bearing triple‐negative breast cancer patient‐derived xenograft (PDX) tumors. (m) Treatment scheme. (n) Tumor growth curves. IgG vs. TIGIT antibody, *p = 0.0343; IgG vs. PD‐L1 antibody, **p = 0.0013; IgG vs. TIGIT×PD‐L1 BsAb, ****p < 0.0001. (o) Kaplan–Meier survival curves. IgG vs. TIGIT antibody, *p = 0.0263; IgG vs. PD‐L1 antibody, *p = 0.0359; IgG vs. TIGIT×PD‐L1 BsAb, **p = 0.0017. For n and o, *n* = 5–12 mice per group. Data are presented as mean ± s.d. Tumor growth curves were analyzed by two‐way ANOVA with a multiple‐comparisons test. Survival curves were analyzed by log‐rank test. *p < 0.05, **p < 0.01, ***p < 0.001, ****p < 0.0001.

We implanted EO771 cells, which express high endogenous *AARS2*, subcutaneously into C57BL/6 mice and then administered IgG, anti‐TIGIT antibody, anti‐PD‐L1 antibody, or TIGIT×PD‐L1 BsAb by intravenous injection (Figure 7b). Either anti‐TIGIT or anti‐PD‐L1 monotherapy produced a partial antitumor effect, whereas TIGIT×PD‐L1 BsAb more strongly suppressed tumor growth and prolonged survival (Figure 7c,d). In addition, the monotherapies and bispecific antibody showed good safety. Mouse body weight remained stable (Figure S3a), peripheral blood parameters were stable (Figure S3b), and major organs showed no clear pathological changes (Figure S3c). Thus, the TIGIT×PD‐L1 bispecific antibody represents a potential immunotherapeutic approach. To determine whether AARS2 is required for the efficacy of TIGIT×PD‐L1 BsAb, we next implanted *Aars2*‐knockout EO771 cells into mice and treated the mice with the bispecific antibody (Figure 7e). The antibody did not further reduce the volume of *Aars2*‐deficient tumors (Figure 7f) and did not significantly prolong mouse survival (Figure 7g). These results indicate that high tumor AARS2 expression is a key biological marker for the efficacy of TIGIT×PD‐L1 BsAb.

To further define how TIGIT×PD‐L1 BsAb reshapes the tumor immune microenvironment in situ, we performed spatial transcriptomic analysis of tumors treated with PD‐L1 monotherapy or TIGIT×PD‐L1 BsAb (Figure 7h). UMAP dimensionality reduction and cell type annotation showed that both groups contained major cell populations of the tumor microenvironment, including cancer cells, CAFs, tumor‐associated macrophages (TAMs), NK cells, and CD8+ T cells, with distinct spatial distribution patterns (Figure 7i and Figure S3d,e). Differential neighborhood interaction analysis showed that, compared with PD‐L1 monotherapy, TIGIT×PD‐L1 BsAb significantly increased the interaction strength between CD8+ T cells or NK cells and cancer cells. These immune cells showed stronger interactions with cancer cells in a high‐death state, suggesting a possible role in tumor killing (Figure 7j and Figure S3f). Spatial in situ imaging further showed clear changes in the spatial adjacency and distribution patterns between immune cells and cancer cells after combination treatment (Figure 7k). Gene set enrichment analysis showed that lymphocytes in the TIGIT×PD‐L1 BsAb group were significantly enriched for immune effector‐related pathways, including lymphocyte activation, innate immune response, positive regulation of immune response, immune response‐activating signaling pathways, and cell surface receptor‐mediated immune signaling (Figure 7l). These data suggest that TIGIT×PD‐L1 BsAb treatment reshapes the tumor immune microenvironment by strengthening spatial interactions between T/NK cells and cancer cells and by broadly activating immune effector functions.

To further validate the efficacy of the bispecific antibody, we obtained tumor and peripheral blood samples from a patient with triple‐negative breast cancer who had responded poorly to multiple lines of therapy, including PD‐L1 blockade. Peripheral blood mononuclear cells (PBMCs) were transferred into NCG mice to establish humanized immune mice, and tumor tissue was then implanted to generate a PDX model. In this model, mice received intravenous treatment with human‐targeted anti‐TIGIT antibody, anti‐PD‐L1 antibody, or TIGIT×PD‐L1 BsAb (Figure 7m). Consistent with the clinical observation, anti‐PD‐L1 monotherapy showed limited antitumor efficacy in this model. In contrast, TIGIT×PD‐L1 BsAb significantly reduced PDX tumor growth and prolonged mouse survival (Figure 7n,o). The human tumor‐directed TIGIT×PD‐L1 bispecific antibody also showed good safety, with stable mouse body weight (Figure S3g). Overall, the TIGIT×PD‐L1 bispecific antibody shows favorable efficacy and safety and may serve as a potential next‐generation immunotherapy.

We also searched for other potential therapies targeting CD112. HSV‐2‐based oncolytic viruses, such as FusOn‐H2, have been developed as promising oncolytic platforms [48, 49]. Because CD112/NECTIN‐2 can serve as an entry receptor for HSV‐2 and supports HSV‐2‐based oncolytic virus infection and spread in CD112‐positive tumor cells [50], AARS2‐mediated CD112 stabilization may influence tumor responsiveness to HSV‐2 oncolytic virotherapy. We implanted control gNC or *Aars2*‐knockout EO771 cells subcutaneously into C57BL/6 mice and administered HSV‐2 oncolytic virus by intratumoral injection (Figure S4a). HSV‐2 oncolytic virus significantly suppressed the growth of control EO771 tumors. *Aars2*‐knockout EO771 tumors already grew slowly, and combination with HSV‐2 maintained a low tumor burden and further improved the survival trend (Figure S4b,c). During treatment, mouse body weight remained stable in all groups (Figure S4d), and peripheral blood counts and major organs showed no clear pathological changes (Figure S4e,f). These results suggest that HSV‐2 oncolytic virotherapy may also be a potential treatment option for tumors with high AARS2 expression.

## 3 Discussion

Tumor metabolic reprogramming, driven by aerobic glycolysis (the Warburg effect), generates massive lactate accumulation that reaches 10–30 mM in the tumor microenvironment (TME) [34, 51, 52, 53]. Conventionally regarded as a metabolic waste product, lactate has been recognized to acidify the TME and indirectly blunt T cell and NK cell activation [13, 14, 15]. The discovery that lactate serves as a direct chemical substrate for protein lactylation—a newly identified post‐translational modification—fundamentally reshaped this view, revealing that lactate can actively reprogram non‐histone protein function beyond passive environmental acidification [53, 54, 55]. The subsequent identification of AARS1 and AARS2 as intracellular L‐lactate sensors and lactyltransferases provided the enzymatic basis for this metabolic–epigenetic coupling [19, 20]. While AARS1 has been shown to promote tumorigenesis primarily through cell‐intrinsic mechanisms, AARS2‐mediated pro‐tumorigenic activity is strictly contingent upon an intact immune microenvironment. This functional dichotomy establishes that AARS2, rather than AARS1, serves as the critical lactyltransferase bridging tumor lactate metabolism to immune evasion, elevating lactate from a passive immunosuppressive metabolite to an active signaling molecule that is enzymatically harnessed by tumor cells to orchestrate immune resistance.

The competitive occupancy of lysine residues by mutually exclusive post‐translational modifications represents a fundamental principle governing protein stability. Prior studies have established that methylation, palmitoylation, and succinylation can protect proteins from ubiquitin‐dependent degradation by blocking E3 ligase recognition [56, 57, 58, 59]. Recent work has extended this paradigm to lactylation: RHOA K162 lactylation competitively antagonizes KCTD13‐mediated poly‐ubiquitination to enhance oncogenic protein stability [60]; NPM1 K257 lactylation suppresses MDM2‐dependent ubiquitination [61]; and tau K331 lactylation inhibits poly‐ubiquitination to promote pathological accumulation [11, 62]. However, whether this lactylation‐ubiquitination antagonism operates on immune checkpoint proteins to regulate their membrane abundance and immunosuppressive efficacy has remained entirely unknown. Our work reveals that AARS2‐mediated CD112 lactylation at lysine 412 establishes a previously unrecognized molecular switch on an immune checkpoint ligand. By catalyzing K412 lactylation, AARS2 impedes the E3 ubiquitin ligase UBE3C, blocking the assembly of K48‐ and K63‐linked poly‐ubiquitin chains and preventing subsequent endosome–lysosomal degradation. This finding extends the lactylation–ubiquitination crosstalk paradigm from oncogenic and neuronal proteins to the immunoregulatory proteome, and demonstrates that metabolic cues can directly dictate the surface density of an immunosuppressive ligand through lysosomal fate control.

Given that AARS2 was previously reported to lactylate cGAS and suppress the cGAS‐STING innate immune pathway 19, an alternative hypothesis would posit that AARS2 promotes immune escape indirectly by dampening interferon signaling and preventing cytotoxic lymphocyte recruitment. However, our analyses indicate that AARS2‐mediated immune evasion operates primarily through the CD112 axis rather than the cGAS‐STING pathway in the examined contexts. By systematically excluding this alternative mechanism, we firmly establish CD112 as the principal downstream effector through which AARS2 exerts its immune‐regulatory function, highlighting context‐dependent substrate specificity as a key feature of lactyltransferase biology.

TIGIT has emerged as a next‐generation immune checkpoint target, and preclinical studies suggested that dual TIGIT and PD‐1/PD‐L1 blockade could synergistically restore antitumor immunity [46, 47, 63]. Early‐phase trials such as CITYSCAPE reported promising efficacy signals in PD‐L1‐positive non‐small cell lung cancer [64]. However, subsequent clinical development of TIGIT‐directed therapies has shown inconsistent efficacy across tumor types and treatment settings. For example, the SKYSCRAPER‐14 trial in hepatocellular carcinoma missed its progression‐free survival endpoint [65], and several anti‐TIGIT agents—including vibostolimab, belrestotug, and ociperlimab—have faced discontinuation or development setbacks due to limited efficacy or safety concerns [66, 67, 68, 69]. These mixed clinical outcomes suggest that broad TIGIT blockade without biomarker‐based patient selection may be insufficient to produce consistent clinical benefit. Notably, positive signals have been observed in specific contexts, such as the SKYSCRAPER‐08 trial in esophageal squamous cell carcinoma and the EDGE‐Gastric study with the Fc‐silent antibody domvanalimab [70], suggesting that TIGIT‐directed therapies may be effective only in discrete molecular subgroups. Our findings provide a metabolic rationale for this hypothesis: tumors with elevated AARS2 activity exhibit stabilized CD112 surface expression, creating a ligand‐enriched context in which TIGIT blockade would be most efficacious. Thus, AARS2 expression or CD112 lactylation status may serve as predictive biomarkers to stratify patients who would benefit from TIGIT‐directed combination immunotherapy, offering a framework for biomarker‐driven clinical trial design that moves beyond one‐size‐fits‐all checkpoint blockade.

In parallel, CD112 functions as the high‐affinity entry receptor for HSV‐2, providing a natural tropism mechanism for oncolytic virotherapy [71]. Oncolytic HSV‐based vectors have shown promise in preclinical models across multiple malignancies [72, 73, 74]. Our observation that AARS2 activity stabilizes CD112 at the plasma membrane suggests that tumors with elevated AARS2 signaling may present enriched CD112 surface density, rendering them selectively vulnerable to HSV‐2 oncolytic infection. This establishes a metabolism‐informed viral targeting strategy, wherein tumor metabolic features are converted into guidance determinants for oncolytic virus therapy.

Several limitations of this study should be acknowledged. First, the functional characterization of the AARS2‐CD112 axis was performed in selected tumor models, and its generalizability across diverse cancer types requires further validation. Second, although our genetic and pharmacological data support a steric hindrance model for lactylation‐mediated UBE3C exclusion, direct structural evidence—such as cryo‐EM or crystallographic analysis of the AARS2‐CD112‐UBE3C complex—remains to be obtained. Third, the development of selective AARS2 enzymatic inhibitors that spare AARS1 activity poses a significant chemical biology challenge. Finally, the clinical utility of AARS2 expression or CD112 K412 lactylation levels as predictive biomarkers awaits prospective validation in patient cohorts.

In summary, our study reveals that tumor cells exploit Warburg effect‐derived lactate to fuel AARS2‐mediated CD112 lactylation. This modification competitively antagonizes UBE3C‐dependent ubiquitination and subsequent lysosomal degradation, thereby sustaining CD112 surface expression to simultaneously disable both CD8+ T cell and NK cell surveillance. By illuminating a regulatory axis that couples metabolic reprogramming to post‐translational modification and immune checkpoint stability, this work provides a mechanistic foundation for targeting the AARS2‐CD112 lactylation pathway and for stratifying patients who may benefit from TIGIT‐directed immunotherapy or oncolytic virotherapy. These findings are summarized in the proposed working model (Figure 8).

**FIGURE 8:**
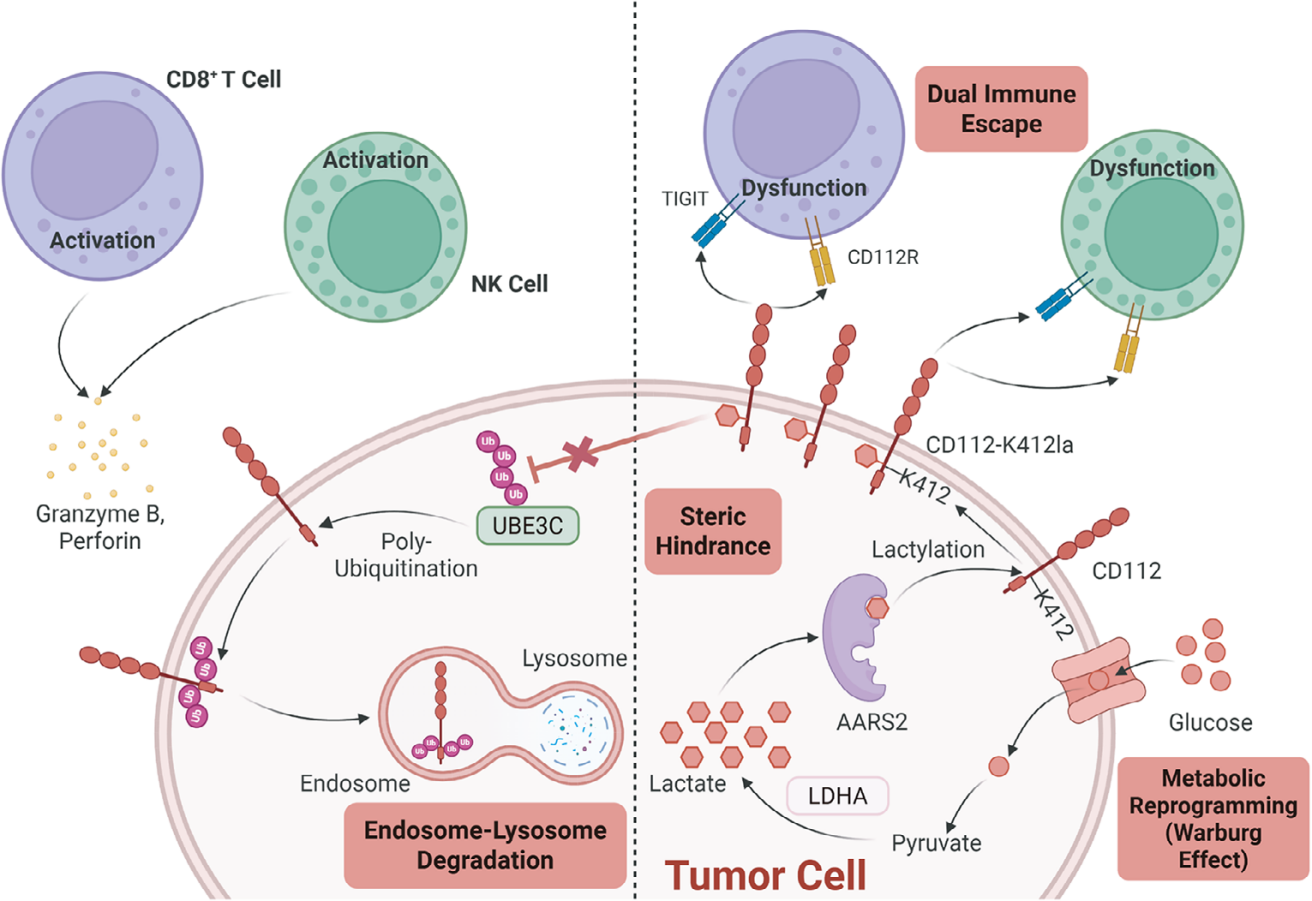
Working model of the AARS2–CD112 lactylation axis in tumor immune escape tumor metabolic reprogramming increases LDHA‐dependent lactate production, which activates the lactyltransferase function of AARS2. AARS2 catalyzes CD112 lactylation at K412, blocking UBE3C‐mediated polyubiquitination and endosome–lysosome degradation of CD112. This process stabilizes CD112 on the tumor cell surface, enabling CD112 to engage TIGIT and CD112R on CD8+ T cells and NK cells. As a result, cytotoxic lymphocyte activation is suppressed, leading to tumor escape from both adaptive and innate immune surveillance.

## 4 Methods

### 4.1 Reagents and Antibodies

Western blotting and immunoprecipitation were performed using primary antibodies at 1:1000 dilution unless otherwise specified. The following antibodies were used: anti‐STING (13647), anti‐phospho‐STING S366 (43499), anti‐phospho‐IRF3 S396 (4947), and anti‐phospho‐STAT1 Y701 (9167) from Cell Signaling Technology; anti‐TBK1 (ab40676), anti‐phospho‐TBK1 S172 (ab109272), and anti‐ubiquitin (ab134953) from Abcam; anti‐IRF3 (sc‐33641) from Santa Cruz Biotechnology; anti‐AARS2 (A25997), anti‐Myc tag (AE070), anti‐STAT1 (A19563), and anti‐tubulin (AC012) from ABclonal; anti‐CD112 (27171‐1‐AP) and anti‐UBE3C (12333‐1‐AP) from Proteintech; anti‐pan‐lysine lactylation (PTM‐1401) from PTM BIO; anti‐β‐actin (A2228, 1:5000) and anti‐FLAG (F3165, 1:5000) from Sigma–Aldrich; and anti‐HA (TA180128) from OriGene.

Flow cytometry antibodies were used at 1:200 dilution unless otherwise specified. Anti‐CD16/CD32 (101301), anti‐mouse APC/Cyanine7‐CD3 (100222), anti‐mouse APC‐CD8 (162306), anti‐mouse PE‐granzyme B (372208), anti‐mouse Brilliant Violet 650‐IFN‐γ (505832), anti‐mouse APC/Cyanine7‐NKp46 (137646), and Tag‐it Violet proliferation and cell tracking dye (425101) were purchased from BioLegend. Anti‐mouse PerCP‐Cy5.5‐CD45 (45‐0451‐82) and anti‐mouse FITC‐TNF‐α (11‐7349‐82) were purchased from Invitrogen. Propidium iodide (PI; HY‐D0815) was purchased from MedChemExpress, and 7‐AAD (A1310) was purchased from Thermo Fisher Scientific.

For in vivo studies, anti‐mouse CD8α (BE0223), anti‐mouse NK1.1 (BE0036), and isotype control IgG2b (BE0090) were purchased from Bio X Cell. The TIGIT×PD‐L1 bispecific antibody was designed, expressed, and purified in‐house as described below. 2‐DG (HY‐13966), oxamate (HY‐W013032A), ARC155858 (HY‐13248), AZD3965 (HY‐12750), sodium L‐lactate (HY‐W040233), β‐alanine (HY‐W012804), cycloheximide (CHX; HY‐12320), NH4Cl (HY‐Y0299), MG132 (HY‐13259), 3‐MA (HY‐19312), and bafilomycin A1 (HY‐100558) were obtained from MedChemExpress.

### 4.2 Cell Culture

HEK293T, B16‐F10, EO771, Panc02, MDA‐MB‐231, and A375 cells were obtained from authenticated commercial sources and cultured in DMEM or RPMI‐1640 medium (Gibco) supplemented with 10% fetal bovine serum (Biochannel) and 1% penicillin–streptomycin. Cells were maintained at 37°C in a humidified incubator containing 5% CO2. Cell lines were authenticated by short tandem repeat profiling where applicable, confirmed to be free of mycoplasma contamination, and used within 6 months after thawing.

### 4.3 Human Samples, PBMC Isolation, and Lymphocyte Culture

Human peripheral blood samples and patient‐derived tumor samples were collected under protocols approved by the Medical Ethics Committee of Union Hospital, Tongji Medical College, Huazhong University of Science and Technology (approval no. [2026] (0139)), and written informed consent was obtained from each donor or patient.

### 4.4 Generation of MDA‐MB‐231 Lysate‐Primed human CD8+ T Cells

Peripheral blood mononuclear cells were isolated from healthy donor blood by Ficoll density‐gradient centrifugation. CD14^+^ monocytes and CD8+ T cells were purified from the same donor using magnetic‐selection kits.

CD14^+^ monocytes were seeded at 1 × 10^6^ cells/mL and cultured in RPMI‐1640 containing 10% heat‐inactivated human AB serum, 25 ng/mL recombinant human GM‐CSF, and 5 ng/mL recombinant human IL‐4. On day 3, half of the culture medium was replaced with fresh medium containing GM‐CSF and IL‐4 at the same final concentrations. Thus, GM‐CSF and IL‐4 were supplied twice, on days 0 and 3. On day 6, immature dendritic cells were matured for 48 h with 100 ng/mL LPS and 500 IU/mL recombinant human IFN‐γ. These differentiation and maturation conditions were adapted from a published tumor‐lysate‐pulsed dendritic‐cell protocol [75].

MDA‐MB‐231 cells were harvested during logarithmic growth, washed extensively with PBS, and subjected to five consecutive freeze–thaw cycles. Insoluble debris was removed by centrifugation, and the protein concentration of the clarified lysate was determined using a BCA assay. Mature dendritic cells were incubated with MDA‐MB‐231 lysate for 24 h at a concentration of 200 µg lysate protein per 1 × 10^6^ dendritic cells per milliliter. After loading, dendritic cells were washed to remove residual soluble lysate. Five freeze–thaw cycles and a loading dose of 200 µg protein per 1 × 10^6^ dendritic cells have been used for breast tumor lysate‐pulsed dendritic cells [76].

Purified autologous CD8+ T cells were cocultured with lysate‐loaded dendritic cells at a CD8+ T‐cell‐to‐dendritic‐cell ratio of 5:1. The cultures were maintained in X‐VIVO 15 or RPMI‐1640 containing 10% heat‐inactivated human AB serum. Recombinant human IL‐2 at 50 IU/mL and IL‐7 at 20 ng/mL were added on culture days 3, 5, and 7.

On day 7, viable CD8+ T cells were collected, washed, and restimulated with freshly prepared MDA‐MB‐231 lysate‐loaded autologous dendritic cells at the same 5:1 ratio. Fresh IL‐2 at 50 IU/mL and IL‐7 at 20 ng/mL were added again on days 10, 12, and 14. Therefore, each cytokine was supplied a total of six times during the 14‐day expansion period. Repeated weekly stimulation with lysate‐loaded dendritic cells and intermittent supplementation with IL‐2 and IL‐7 are established approaches for expanding tumor‐lysate‐reactive T cells.

Cells were maintained at approximately 0.5–1 × 10^6^ cells/mL, and the medium was adjusted as required to maintain this density. On day 14, CD8+ T cells were re‐isolated from the co‐culture by magnetic selection to remove residual dendritic cells and other non‐CD8^+^ populations, washed, and analyzed by flow cytometry. Only preparations with greater than 90% viability and greater than 90% CD3^+^CD8^+^ purity were used for adoptive transfer.

### 4.5 Isolation and Expansion of Primary human NK Cells

Primary human NK cells were isolated from donor PBMCs by negative immunomagnetic selection and cocultured with irradiated (100 Gy) K562 cells expressing membrane‐bound IL‐21 and 4‐1BB ligand (K562‐mbIL21‐4‐1BBL) at a feeder‐to‐NK ratio of 1.5:1 in NK MACS medium containing 5% human platelet lysate and 50 U/mL recombinant human IL‐2. Fresh IL‐2 was replenished every 2–3 days, and cell density was maintained at 0.5–1.5 × 10^6^ cells/mL. On day 7, NK cells were restimulated with a fresh batch of irradiated K562‐mbIL21‐4‐1BBL feeder cells. Cells were harvested on day 14, and residual feeder cells were removed by magnetic depletion when necessary. This feeder‐based protocol yields highly activated primary NK cells and is widely used in clinical‐grade NK‐cell manufacturing [77]. Before transfer, viability and purity were evaluated by flow cytometry. Only preparations with greater than 90% viability and greater than 90% CD3^−^CD56^+^ purity were used.

### 4.6 Mouse OT‐I CD8+ T Cell Isolation and Activation

OT‐I CD8+ T cells were isolated from the spleens and lymph nodes of C57BL/6‐Tg(TcraTcrb)1100Mjb/J mice. Spleens were mechanically dissociated and passed through a 70‐µm cell strainer. Red blood cells were lysed for 3 min using Red Blood Cell Lysis Buffer (Solarbio), and the reaction was stopped by adding RPMI‐1640 medium supplemented with 10% fetal bovine serum. Cells were resuspended at 2 × 10^6^ cells/mL in RPMI‐1640 complete medium containing 5 µg/mL OVA257‐264 peptide (RP10611CN, GenScript), 40 µM 2‐mercaptoethanol (M3148, Sigma–Aldrich), and penicillin–streptomycin, and were cultured at 37°C with 5% CO2. Recombinant mouse interleukin‐2 (2 ng/mL; 402‐ML, R&D Systems) was added on day 2, and fresh medium was supplied daily. On day 4, activated OT‐I cells were purified using the EasySep Mouse CD8+ T Cell Isolation Kit (19853, STEMCELL Technologies).

### 4.7 Animal Studies

C57BL/6J wild‐type mice and female NCG mice (NOD/ShiLtJGpt‐Prkdcem26Cd52 Il2rgem26Cd22/Gpt) were purchased from GemPharmatech. Mice were 6–8 weeks of age at the start of experiments and were randomly assigned to experimental groups. Animals were housed under specific pathogen‐free conditions at approximately 23°C and 50% humidity with a 12‐h light/12‐h dark cycle. All animal procedures were approved by the Institutional Animal Care and Use Committee of Tongji Medical College, Huazhong University of Science and Technology (HUST‐IACUC‐2025‐0016).

For mouse subcutaneous tumor models, EO771 cells (1 × 10^6^), B16‐F10 cells (5 × 10^5^), or Panc02 cells (1 × 10^6^) were injected subcutaneously into C57BL/6J mice. Tumor length and width were measured every 2–3 days, and tumor volume was calculated as length × width^2^ × 0.5. Mice were euthanized when tumor volume reached 2000 mm^3^ or when humane endpoints were met.

### 4.8 Human Lymphocyte Adoptive‐Transfer Model

Female NCG mice aged 6–8 weeks were orthotopically implanted in the mammary fat pad with 1 × 10^6^ control or AARS2‐knockout MDA‐MB‐231 cells. When tumors reached approximately 50 mm^3^, mice were stratified by tumor volume and randomized into the indicated treatment groups.

Mice received the following treatments by tail‐vein injection on treatment days 0 and 7: 6 × 10^6^ MDA‐MB‐231 lysate‐primed CD8+ T cells; 6 × 10^6^ expanded primary NK cells; 6 × 10^6^ CD8+ T cells plus 6 × 10^6^ NK cells; vehicle without immune cells.

Recombinant human IL‐2 was administered intraperitoneally at 5000 IU per mouse once daily for four consecutive days after each transfer. IL‐2 was therefore administered on days 0, 1, 2, and 3, and again on days 7, 8, 9, and 10, for a total of eight injections per mouse. All immune‐cell‐transfer groups received the same IL‐2 regimen. Repeated transfer and short‐course IL‐2 support are consistent with published human lymphocyte‐transfer studies in MDA‐MB‐231‐bearing immunodeficient mice.

For patient‐derived xenograft (PDX) studies, tumor tissue and paired peripheral blood samples were obtained from the same patient with triple‐negative breast cancer under approved human‐subject protocols. PBMCs were isolated by Ficoll density‐gradient centrifugation, and preparations with a viability of at least 90% were used. Each mouse received 5 × 10^6^ viable autologous PBMCs by tail‐vein injection without irradiation or other preconditioning, and tumor fragments derived from the same patient were implanted subcutaneously. Human immune‐cell engraftment was assessed in peripheral blood on days 7 and 14 after PBMC administration by flow cytometry. Human leukocytes were identified as human CD45^+^ mouse CD45^−^ cells, and mice with at least 2% circulating human CD45^+^ cells at both time points were considered successfully humanized. When tumors became palpable and reached the size indicated in the figure legends, successfully engrafted mice were stratified according to tumor volume, circulating human CD45^+^ cell frequency, and body weight, and were randomly assigned to receive IgG control, anti‐TIGIT antibody, anti‐PD‐L1 antibody, or TIGIT×PD‐L1 bispecific antibody. Tumor dimensions and body weight were recorded every 3 days. Xenogeneic graft‐versus‐host disease was prospectively monitored by assessing body‐weight loss, posture, activity, fur condition, skin integrity, and diarrhea. Day 15 was predefined as the primary tumor‐growth evaluation time point rather than the termination of the animal experiment. After completion of the predefined dosing period, tumor monitoring and survival follow‐up continued until individual mice reached the predefined humane endpoints. At the experimental endpoint, the heart, liver, spleen, lungs, and kidneys were collected and subjected to hematoxylin and eosin staining to evaluate inflammatory‐cell infiltration, tissue injury, necrosis, and other pathological alterations potentially associated with xenogeneic GVHD or treatment‐related toxicity.

For in vivo immune cell depletion, mice bearing EO771 tumors were treated when tumors reached approximately 100 mm^3^, typically 3–5 days after implantation. Mice received isotype‐matched IgG, anti‐CD8α, anti‐NK1.1, or both depletion antibodies every 3 days. Depletion efficiency was verified by flow cytometry of peripheral blood or tumor tissues.

For antibody treatment experiments, mice with established tumors were randomized when tumors reached approximately 100 mm^3^ and were treated intravenously with IgG control, anti‐TIGIT antibody, anti‐PD‐L1 antibody, or TIGIT×PD‐L1 bispecific antibody at the doses and schedules indicated in the figure legends. Body weight was monitored throughout treatment. Peripheral blood and major organs were collected for safety evaluation where indicated.

For HSV‐2 oncolytic virotherapy, mice bearing wild‐type or *Aars2*‐knockout EO771 tumors were treated with HSV‐2 by intratumoral injection when tumors reached approximately 100 mm^3^. Each injection contained 2 × 10^6^ plaque‐forming units (pfu) of virus. Injections were given every other day for a total of three injections. For survival studies, mice were euthanized when the mean tumor diameter exceeded 15 mm, tumor volume exceeded 2000 mm^3^, or tumor ulceration reached the institutional humane endpoint.

### 4.9 Constructs

Mammalian expression plasmids encoding FLAG‐, HA‐, or Myc‐tagged human *AARS2*, mouse *Aars2*, human *CD11*2, human *UBE3C*, human *TRIM4*, and human *SMURF1* were generated using standard molecular biology methods. AARS2 lactyltransferase‐defective mutants (R110A, W210A, and G269A) and CD112 lysine‐to‐arginine mutants (K407R, K412R, K414R, K436R, and K528R) were generated by site‐directed mutagenesis. All constructs were confirmed by DNA sequencing. sgRNAs targeting human *AARS2*, human *CD112*, human *UBE3C*, mouse *Aars1*, mouse *Aars2*, and mouse *Cd112* were cloned into BsmBI‐digested lentiCRISPRv2‐puro or lentiCRISPRv2‐blast vectors (Addgene). For *AARS2* knockdown, two independent short hairpin RNAs targeting human *AARS2* (*shAARS2*#1 and *shAARS2*#2) were designed and cloned into the pLKO.1‐puro lentiviral vector, and the resulting constructs were verified by DNA sequencing before lentiviral packaging and stable cell line generation.

The following human sgRNA sequences were used:

g*AARS2*#1: 5′‐AGCAAACTGGGGTCGCCGCG‐3′;

g*AARS2*#2: 5′‐CCAACTTCTACGCAGAACAG‐3′;

g*CD112*#1: 5′‐ACACTCACAGCGTACAGAGA‐3′;

g*CD112*#2: 5′‐AGCTGGGACCCATCTTAGGG‐3′;

g*UBE3C*#1: 5′‐CATTCAGAAAGGGCTCGTAA‐3′;

g*UBE3C*#2: 5′‐CTCAGACACAGACAGCGGCG‐3′.

The following mouse sgRNA sequences were used:

g*Aars1*#1: 5′‐CTTCAGCAATCAATCCAGTG‐3′;

g*Aars1*#2: 5′‐TGTTATACTGGATGAACACG‐3′;

g*Aars2*#1: 5′‐AGGACGCCATAACGACCTGG‐3′;

g*Aars2*#2: 5′‐CTACACTCTTCACCCCAACG‐3′;

g*Cd112*#1: 5′‐TCCCAACGGTACCCGCAGGG‐3′;

g*Cd112*#2: 5′‐GCGGGTACGAGTGCTTCCCG‐3′.

The following human shRNA sequences were used:

sh*AARS2*#1: 5′‐CCATCATACCTTCTTTGAAAT‐3′;

sh*AARS2*#2: 5′‐CCTGGTCTTCATGCAACACAA‐3′.

All sgRNA and shRNA oligonucleotides were synthesized by Beijing Tsingke Biotech Co., Ltd. (Beijing, China).

### 4.10 Cell Transfection and Stable Cell Line Generation

HEK293T cells were transfected at 50%–70% confluence in 10‐cm dishes or six‐well plates using calcium phosphate precipitation. Cells were collected 18–36 h after transfection for immunoblotting, immunoprecipitation, ubiquitination assays, lactylation assays, or mass spectrometry.

For lentivirus production, HEK293T cells were co‐transfected with the target lentiviral vector and packaging plasmids. Viral supernatants were collected 48 h after transfection, filtered through a 0.45‐µm filter (Millipore), and used to infect target cells in the presence of 8 µg/mL polybrene. Infected cells were selected with puromycin or blasticidin for at least 6 days. OVA‐expressing tumor cells were generated by lentiviral transduction with an ovalbumin expression vector followed by puromycin selection for at least 6 days.

### 4.11 Antibody Purification

The TIGIT×PD‐L1 bispecific antibody was designed using a heterodimeric Fc strategy based on the classical knobs‐into‐holes principle, with the KiHss‐AkKh Fc variant used to promote preferential heavy‐chain heterodimerization. The single‐chain variable fragment (scFv) of anti‐PD‐L1 was fused to the knob‐mutant Fc arm, whereas the anti‐TIGIT scFv was fused to the hole‐mutant Fc arm. The two expression plasmids were transiently co‐transfected into FreeStyle 293‐F cells according to the manufacturer's protocol. Culture supernatants were harvested after expression and clarified by centrifugation and filtration. The TIGIT×PD‐L1 bispecific antibody was first purified by Protein A affinity chromatography and then buffer‐exchanged into PBS using 50‐kDa centrifugal ultrafiltration devices. To remove aggregates and high‐molecular‐weight impurities, the antibody preparation was further purified by size‐exclusion chromatography on a Superdex 200 pg column. The purity and homogeneity of the purified antibody were assessed by SDS–PAGE under reducing and non‐reducing conditions. The matched anti‐PD‐L1 and anti‐TIGIT monoclonal antibodies, both carrying a human IgG1 Fc backbone, were expressed and purified using the same procedure.

### 4.12 Isolation of Tumor‐Infiltrating Immune Cells

Subcutaneous tumors were collected aseptically, minced with sterile scissors, and digested with collagenase IV (1 mg/mL; 17104019, Gibco) and DNase I (M0303S, NEB). Single‐cell suspensions were prepared by passing the digested tissue through 70‐µm cell strainers. Tumor‐infiltrating lymphocytes (TILs) were enriched by density‐gradient centrifugation using Lymphocyte Separation Medium (17089101, Cytiva). For intracellular cytokine staining, cells were stimulated in complete medium containing PMA (20 ng/mL), ionomycin (1 µg/mL), brefeldin A (1:1000), and monensin (1:1000) at 37°C. Cells were stained for surface markers, fixed, permeabilized using the eBioscience Intracellular Fixation and Permeabilization Buffer Set (88‐8824‐00, Thermo Fisher Scientific), and stained with intracellular antibodies. Flow cytometry was performed on a CytoFLEX LX flow cytometer, and data were analyzed using FlowJo V10.

### 4.13 Flow Cytometry

Flow cytometry was performed as previously described [78, 79]. Cells were subjected to staining with the indicated antibodies for 30 min at 4°C. The cells were acquired with a CytoFLEX LX flow cytometer (Beckman Coulter). The data were processed and analyzed using FlowJo V10.

### 4.14 In Vitro CD8+ T‐Cell and NK‐Cell Cytotoxicity Assays

Tumor‐cell killing was assessed by prelabeling target tumor cells with CellTrace, followed by propidium iodide (PI) staining after coculture. Target tumor cells were labeled according to the manufacturer's instructions, washed, and cocultured with unlabeled effector cells at the effector‐to‐target ratios indicated in the figure legends. Human CD8^+^ T‐cell killing assays were performed for 18–24 h, whereas NK‐cell killing assays were performed for 4 h. For antigen‐specific killing assays, CellTrace‐labeled EO771‐OVA tumor cells were cocultured with activated OT‐I CD8^+^ T cells.

After coculture, cells were stained with PI and analyzed by flow cytometry. Dead target tumor cells were defined as CellTrace^+^PI^+^ events, and tumor‐cell death was calculated as the percentage of PI^+^ cells within the CellTrace^+^ target‐cell population. Flow‐cytometric data were analyzed using FlowJo v10.

For lactate metabolism intervention experiments, tumor cells were treated with 2‐DG (10 mM, 24 h), oxamate (20 mM, 24 h), AR‐C155858 (100 nM, 4 h), or AZD3965 (100 nM, 4 h) before coculture with effector cells.

### 4.15 Quantitative PCR With Reverse Transcription

Total RNA was extracted using RNAiso Plus (Takara). One microgram of total RNA was reverse‐transcribed using HiScript II Q RT SuperMix for qPCR with gDNA wiper (Vazyme). Quantitative PCR was performed using ChamQ Universal SYBR qPCR Master Mix (Vazyme) on a real‐time PCR system. Relative mRNA expression was calculated using the 2^−ΔΔCt^ method and normalized to the indicated housekeeping gene. The following primers were used:

Ifnb1 Forward: 5’‐TCCTGCTGTGCTTCTCCACCACA‐3’;

Ifnb1 Reverse: 5’‐AAGTCCGCCCTGTAGGTGAGGTT‐3’;

Cxcl10 Forward: 5’‐ATCATCCCTGCGAGCCTATCCT‐3’;

Cxcl10 Reverse: 5’‐GACCTTTTTTGGCTAAACGCTTTC‐3’;

Isg56 Forward: 5’‐ACAGCAACCATGGGAGAGAATGCTG‐3’;

Isg56 reverse: 5’‐ACGTAGGCCAGGAGGTTGTGCAT‐3’.

All primers were synthesized by Sangon Biotech (Shanghai) Co., Ltd. (Shanghai, China).

### 4.16 Immunofluorescence Imaging

Immunofluorescence was performed as previously described [80, 81]. For IF staining of cultured cells, cells grown on glass coverslips were washed with PBS, fixed with 4% paraformaldehyde at room temperature for 20 min, and permeabilized with 0.1% Triton X‐100 in PBS for 30 min. After blocking with 5% BSA, samples were incubated with primary antibodies at 4°C overnight, followed by corresponding secondary antibodies for 1 h at 37°C. Following three washes with PBS containing 0.1% Tween‐20, nuclei were counterstained with 4′,6‐diamidino‐2‐phenylindole (DAPI) (C0065, Solarbio). Images were acquired using laser scanning confocal microscopy (Leica).

For IF staining of tumor tissue, samples were processed, embedded in paraffin, and sectioned at 4 µm. Paraffin sections were then deparaffinized and hydrated using the following steps: 15 min in xylene twice and 5, 5, 5, and 5 min in 100%, 95%, 75%, and 50% ethanol, respectively. Antigen retrieval was achieved by boiling the sections in 10 mmol/L sodium citrate for 20 min in a microwave oven and cooling for 30 min at room temperature. Sections were permeabilized with 0.4% Triton X‐100 in PBS for 10 min and then blocked with 5% BSA in PBS for 1 h at room temperature.

The sections were incubated with anti‐CD8α (98941, Cell Signaling Technology) and anti‐CD112 (27171‐1‐AP, Proteintech) at 4°C overnight. Subsequently, the sections were stained with Alexa Fluor 488‐ and 647‐conjugated secondary antibodies (A21206, Invitrogen) for 1 h at room temperature. After incubation with DAPI, the slides were mounted using SlowFade Diamond Antifade Mountant (Thermo Fisher Scientific, catalog no. S36967). Images were collected using a fluorescence microscope or confocal microscope, and positive cells or fluorescence intensity were quantified using ImageJ or equivalent image‐analysis software.

### 4.17 Immunoprecipitation and Immunoblot

Immunoblotting was performed as previously described [82]. Cells were lysed in 1 mL of NP‐40 lysis buffer (20 mM Tris‐HCl, 150 mM NaCl, 1 mM EDTA, 1% Nonidet P‐40, 1% Triton X‐100, 10 µg/mL aprotinin, 10 µg/mL leupeptin, and 1 mM phenylmethylsulfonyl fluoride, PMSF). For each immunoprecipitation reaction, a 0.8 mL aliquot of lysate was incubated with 0.5–2 µg of the indicated antibody or control IgG and 30 µL of a 1:1 slurry of Protein‐G Sepharose at 4°C for 3 h. The Sepharose beads were washed three times with 1 mL of lysis buffer containing 0.5 M NaCl. The precipitates were fractionated by SDS‐PAGE, and immunoblotting analysis was performed with the indicated antibodies.

### 4.18 Protein Half‐Life Assay

To measure CD112 protein stability, cells were treated with cycloheximide at 200 µg/mL for the indicated times to block new protein synthesis. Cells were collected at each time point, and CD112 protein levels were measured by Western blotting. Band intensity was quantified and normalized to the loading control to estimate relative CD112 stability.

### 4.19 Mass Spectrometry and Proteome Analysis

For identification of CD112 lactylation sites, HEK293T or MDA‐MB‐231 cells expressing Flag‐tagged CD112 were lysed in NP‐40 lysis buffer supplemented with protease inhibitors. Flag‐CD112 was immunoprecipitated using anti‐Flag M2 affinity gel. Immunoprecipitated proteins were separated by SDS‐PAGE, and gel bands corresponding to CD112 were excised and subjected to in‐gel trypsin digestion. Peptides were desalted using C18 StageTips before LC‐MS/MS analysis.

For CD112 interactome analysis, 293T cells expressing Flag‐tagged CD112 or an empty vector control were lysed in NP‐40 lysis buffer. Flag‐CD112‐associated protein complexes were immunoprecipitated using anti‐Flag M2 affinity gel. Bound proteins were separated by SDS‐PAGE and subjected to in‐gel trypsin digestion. The resulting peptides were desalted using C18 StageTips before LC‐MS/MS analysis.

Peptides were separated on a nanoACQUITY UPLC system (Waters) equipped with a nanoEase M/Z HSS T3 column (1.8 µm, 75 µm × 250 mm) using a 60‐min linear gradient. Mobile phase A consisted of 0.1% formic acid in water, and mobile phase B consisted of 0.1% formic acid in acetonitrile. Eluted peptides were analyzed on a SCIEX ZenoTOF 7600 system operated in positive electrospray ionization mode. For lactylation site mapping and CD112 interactome analysis, data‐dependent acquisition was performed.

Database searching was performed using Proteome Discoverer (v3.1, Thermo Fisher Scientific) with SEQUEST HT and Mascot (v2.8) against the UniProt Homo sapiens reference proteome database (release 2024_04). Search parameters included a precursor mass tolerance of 10 ppm and a fragment mass tolerance of 0.02 Da. Carbamidomethylation of cysteine was set as a fixed modification, whereas oxidation of methionine, protein N‐terminal acetylation, and lysine lactylation (K +72.0211 Da) were set as variable modifications for lactylation site analysis. Peptide‐spectrum matches were filtered using a target‐decoy strategy, with the false discovery rate controlled at <1%. Lactylation site localization was assessed using the ptmRS node, and sites were assigned only when the localization probability exceeded 0.75.

For global proteome comparison between control and *AARS2*‐deficient cells, equal amounts of total protein were extracted using the EasyPep Mini MS Sample Prep Kit (Thermo Fisher Scientific). Proteins were reduced, alkylated, and digested with trypsin/Lys‐C. Peptides were desalted and reconstituted in 0.1% formic acid before LC‐MS/MS analysis.

Peptide separation was performed on a nanoACQUITY UPLC system with a 60‐min linear gradient on a nanoEase M/Z HSS T3 column. Mass spectrometric analysis was conducted on a SCIEX ZenoTOF 7600 system in positive electrospray ionization mode, and data were acquired using the Zeno SWATH DIA workflow. Variable Q1 window sizes were used according to sample complexity.

Raw DIA data were processed in Spectronaut (v20.2, Biognosys) using directDIA mode. Data were searched against the UniProt reference proteome database corresponding to the species origin of each sample. Carbamidomethylation of cysteine was set as a fixed modification, and oxidation of methionine and protein N‐terminal acetylation were set as variable modifications. Identifications were filtered to a false discovery rate of <1% at both precursor and protein levels. Interference correction and cross‐run normalization were applied for protein quantification. Differential protein abundance analysis was performed using limma (v3.60.0), and proteins with |log2 fold change| > 0.5 and adjusted *p* < 0.05 were considered significantly changed.

### 4.20 RNA‐seq

Total RNA was extracted from B16‐F10 cells using RNAiso Plus (Takara). Library preparation and 150‐bp paired‐end sequencing on an Illumina platform were performed by Novogene. Low‐quality reads and adaptor sequences were removed before alignment to the mouse reference genome. Gene‐level counts were generated, normalized, and used for differential gene expression and pathway analyses.

### 4.21 Spatial Transcriptome Analysis

Spatial transcriptome profiling was performed using the Stereo‐seq platform (BGI) on 10 × 10 mm DNA nanoball‐patterned arrays. FFPE tissue sections (5 µm) were mounted onto the chip surface and processed for in situ RNA capture, reverse transcription, and library construction according to the manufacturer's protocol. Paired‐end sequencing was performed on the DNBSEQ‐T10 platform, with read 1 used to decode coordinate identifiers (CID) and molecular identifiers (MID), and read 2 used to sequence cDNA inserts.

Raw data were processed using the Stereo‐seq Analysis Workflow (SAW) pipeline. Read 1 was decoded to extract CID and MID information, and read 2 was aligned to the species‐matched reference genome using STAR (v2.7.11b). GRCh38 was used for human samples, and mm39 was used for mouse samples. Low‐quality and unmapped reads were removed, and uniquely mapped transcripts were collapsed by CID, MID, and gene locus to generate a spatially resolved gene expression matrix. Expression counts were aggregated into 25 µm × 25 µm spatial bins (bin50) to improve transcript coverage per spatial unit.

Downstream analysis was performed using R (v4.4) and Python (v3.12). Data preprocessing, normalization, dimensionality reduction, and clustering were performed using Seurat (v5.1.0), Scanpy (v1.10.4), and Squidpy (v1.6.1). After log‐normalization and selection of highly variable genes, principal component analysis (PCA) and UMAP dimensionality reduction were performed. Cell clusters were annotated using SingleR together with canonical lineage markers. Species‐matched marker genes were used, including Epcam/EPCAM for tumor cells, Cd3e/CD3E and Cd8a/CD8A for CD8+ T cells, Ncr1/NCR1 for NK cells, Adgre1/ADGRE1 for macrophages, and Pdgfra/PDGFRA for fibroblasts and cancer‐associated fibroblasts. Differential neighborhood interaction analysis was performed using Squidpy to compare spatial neighborhood associations between cytotoxic lymphocytes and tumor cells across treatment groups. Gene set enrichment analysis was performed using clusterProfiler (v4.14.0) based on cluster‐specific differentially expressed genes to identify enriched immune effector pathways. Spatial visualization of cell‐type distributions and gene expression was generated using Scanpy, ggplot2 (v3.5.1), and the Seurat spatial plotting framework.

### 4.22 CRISPR‐Screen Analysis

CRISPR screen sequencing data were processed using MAGeCK (v0.5.9.4). FASTQ files were first trimmed to extract sgRNA sequences and then mapped to the corresponding reference sgRNA libraries using the mageck count module. Non‐targeting sgRNAs were used as negative controls for normalization and quality assessment. sgRNA read counts were normalized across samples using the median‐ratio normalization method implemented in MAGeCK.

For each screen, gene‐level enrichment or depletion was calculated using the MAGeCK robust rank aggregation (RRA) algorithm. For the in vivo immune pressure screen, sgRNA abundance was compared between tumors grown under immunocompetent and immunodeficient conditions to identify genes associated with tumor fitness under immune pressure. For the antigen‐specific CD8+ T cell killing screen and the NK cell cytotoxicity screen, sgRNAs depleted from surviving tumor cells after immune cell co‐culture were considered to target genes required for resistance to immune killing. Gene‐level hits were defined using an RRA score < 0.05. Mouse and human gene symbols were converted to species‐matched orthologues before cross‐screen comparison. Candidate dual immune escape genes were defined as genes consistently identified across the in vivo immune pressure screen, the antigen‐specific CD8+ T cell killing screen, and the NK cell cytotoxicity screen.

### 4.23 Statistical Analysis

All statistical analyses were performed using GraphPad Prism (v10.3.1) and R (v4.4.1), as indicated. Data distribution was assessed using the Shapiro–Wilk normality test and Levene's test for homogeneity of variance. For comparisons between two groups with normal distribution and equal variance, a two‐tailed unpaired Student's *t*‐test was applied. For comparisons among three or more groups, one‐way ANOVA with Tukey's or Dunnett's multiple‐comparisons test, or two‐way ANOVA with Tukey's, Dunnett's, or Šídák's multiple‐comparisons test was used as specified in the corresponding figure legends. When normality assumptions were violated, a two‐tailed unpaired Mann–Whitney U test (for two groups) or Kruskal–Wallis test with Dunn's post hoc test (for multiple groups) was employed. For tumor growth curves, two‐way repeated‐measures ANOVA with Šídák's or Bonferroni's multiple‐comparisons test was used. For in vitro cytotoxicity assays across multiple effector‐to‐target ratios, two‐way ANOVA with Tukey's multiple‐comparisons test was applied.

For survival analysis, Kaplan–Meier curves were generated, and statistical significance was determined by the log‐rank (Mantel–Cox) test. Correlation analyses were performed using Spearman's rank correlation test for non‐parametric data or Pearson's correlation test for parametric data. For CRISPR screen analysis, gene‐level significance was determined using the MAGeCK robust rank aggregation (RRA) algorithm, with RRA score < 0.05 considered significant. For RNA‐seq differential expression analysis, DESeq2 (v1.44.0) was used with a threshold of |log_2_ fold change| ≥ 1.5 and Benjamini–Hochberg‐adjusted *p* value < 0.05. For proteomics data, differentially expressed proteins were identified using a moderated *t*‐test (limma, v3.60.0) with |log_2_ fold change| > 0.5 and adjusted *p*‐value < 0.05. For spatial transcriptome data, differentially expressed genes between clusters or treatment groups were identified using the Wilcoxon rank‐sum test implemented in Seurat (v5.1.0), with adjusted *p*‐value < 0.05 (Bonferroni correction).

Unless otherwise specified in figure legends, data are presented as mean ± standard deviation (s.d.) for normally distributed continuous variables or median with interquartile range (IQR) for non‐normally distributed variables. The number of independent biological replicates (*n*) is indicated in each figure legend. For all biochemical experiments, including immunoblotting and immunoprecipitation, at least two independent experiments were performed with similar results. For animal studies, mice were randomly allocated to treatment groups using a random number generator. Investigators were blinded to group allocation during tumor volume measurement, flow cytometry analysis, and histological evaluation. Statistical significance was defined as ^*^ *p* < 0.05, **p < 0.01, ***p < 0.001, ****p < 0.0001; ns, not significant. Exact *p*‐values are provided in the Source Data files where applicable. No data points were excluded from analysis unless pre‐established exclusion criteria were met (e.g., tumor ulceration exceeding 10 mm in diameter or mouse death prior to endpoint).

## Funding

This study was supported, in part, by the National Natural Science Foundation of China grants (82673430 (Z.Wu), 82303179 (Z. Wu), 82404027 (J. Zhou), 82503923 (Z. Xi)), the Natural Science Foundation of Hubei Province grants (2024AFB626 (Z. Wu), 2025AFD800 (F. Dong), 2026AFB530 (Z. Xi)), and the Hubei Province Postdoctoral Innovation Practice Program grant 2023–36 (Z. Wu).

## Author Contributions

Z. Xu conceived the work and designed the experiments with input from J. Zhou, Z. Xi and Z. Wu. Z. Xu and X. Zhang jointly designed and implemented the CRISPR screen analysis pipeline and performed CRISPR screening, spatial transcriptomic analyses and quantitative validation of the AARS2‐CD112 regulatory axis. F. Dong performed the lactylation, ubiquitination, immunoprecipitation, and protein stability assays, and analysed the post‐translational modification mechanism of CD112. N. Shen performed the bioinformatic analyses. Z. Xu and X. Zhang conducted the in vivo tumor models, immune cell depletion experiments, and therapeutic evaluation of TIGIT×PD‐L1 bispecific antibody and HSV‐2 oncolytic virus. Z. Xi and Z. Wu performed flow cytometry quantification of tumor‐infiltrating lymphocytes and interpreted the immune escape mechanism data. J. Zhou supervised the project, secured funding, and revised the manuscript. Z. Xi and Z. Wu provided experimental guidance, designed the therapeutic strategies, and edited the manuscript. All authors contributed to data interpretation, manuscript drafting, and approved the final version.

## Declaration of AI Use

The authors acknowledge the use of Kimi K2 (Moonshot AI) for language polishing, translation, and assistance with literature screening. All AI‐assisted text and literature‐screening outputs were critically reviewed and validated by the authors, who made the final decisions regarding wording, interpretation, and literature inclusion. Draft schematic diagrams were prepared in BioRender with AI‐assisted layout suggestions and were subsequently reviewed and refined by the authors to ensure scientific accuracy. Neither tool was used to generate or analyze research data or to determine the study conclusions. The authors take full responsibility for the accuracy, integrity, and originality of the final manuscript.

## Conflicts of Interest

The authors declare no conflicts of interest.

## Supporting information




**Supporting File 1**: advs78088‐sup‐0002‐SuppMat.docx.

**Supporting File 2**: advs78088‐sup‐0001‐Figure1 WB.pdf.

## Data Availability

All raw data supporting the conclusions of this study, including CRISPR screening datasets, spatial transcriptomic/RNA‐seq profiles, mass spectrometry results, Western blot images, flow cytometry plots, immunohistochemistry results and in vivo animal experiment records, are available from the corresponding authors upon reasonable request.
