# Supplementary material for: CD112 Lactylation Drives Dual Immune Evasion From CD8+ T Cells and NK Cells
Source: Adv Sci (Weinh). 2026 Oct 4:e78088. Online ahead of print. doi: 10.1002/advs.78088 (PMC13634482; doi:10.1002/advs.78088)

Supplementary Material: Uncropped immunoblots from Main Figures

For Figure 4

Fig. 4i

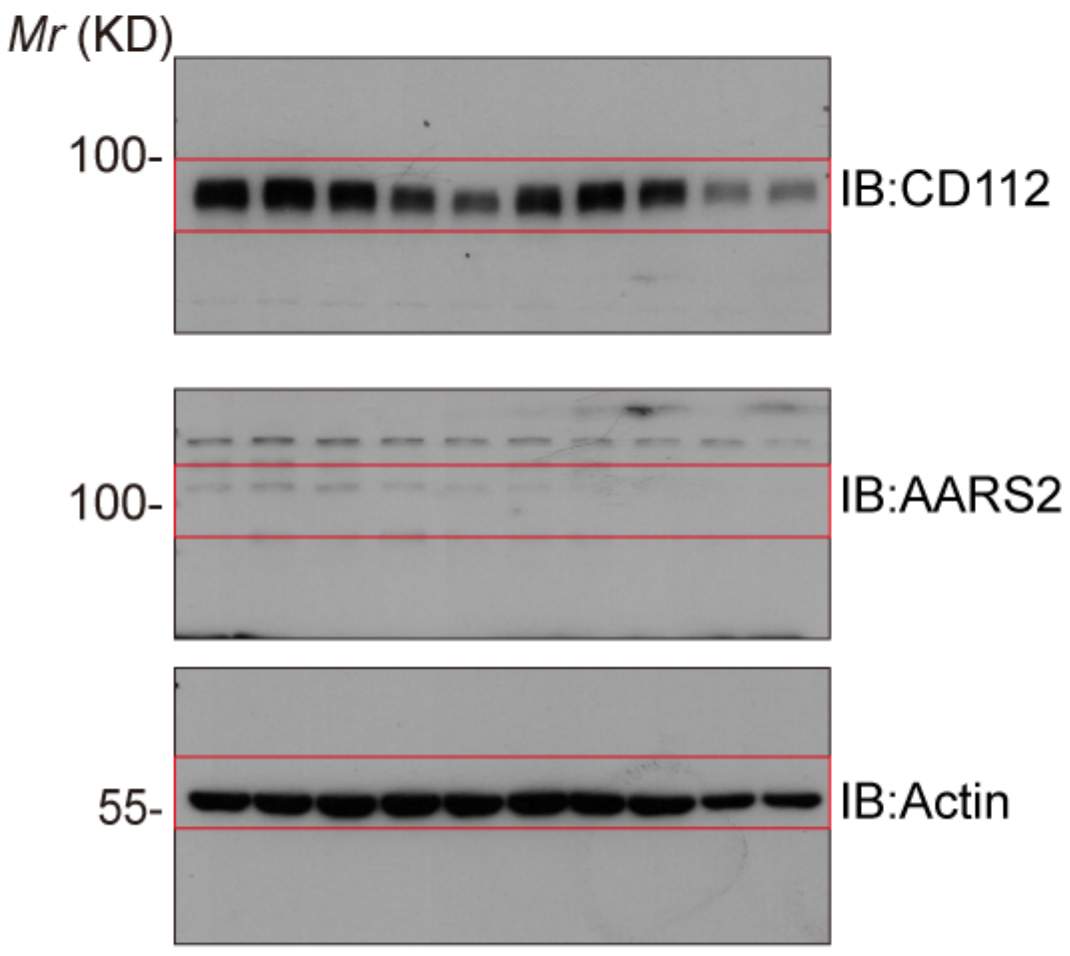

Fig. 4k

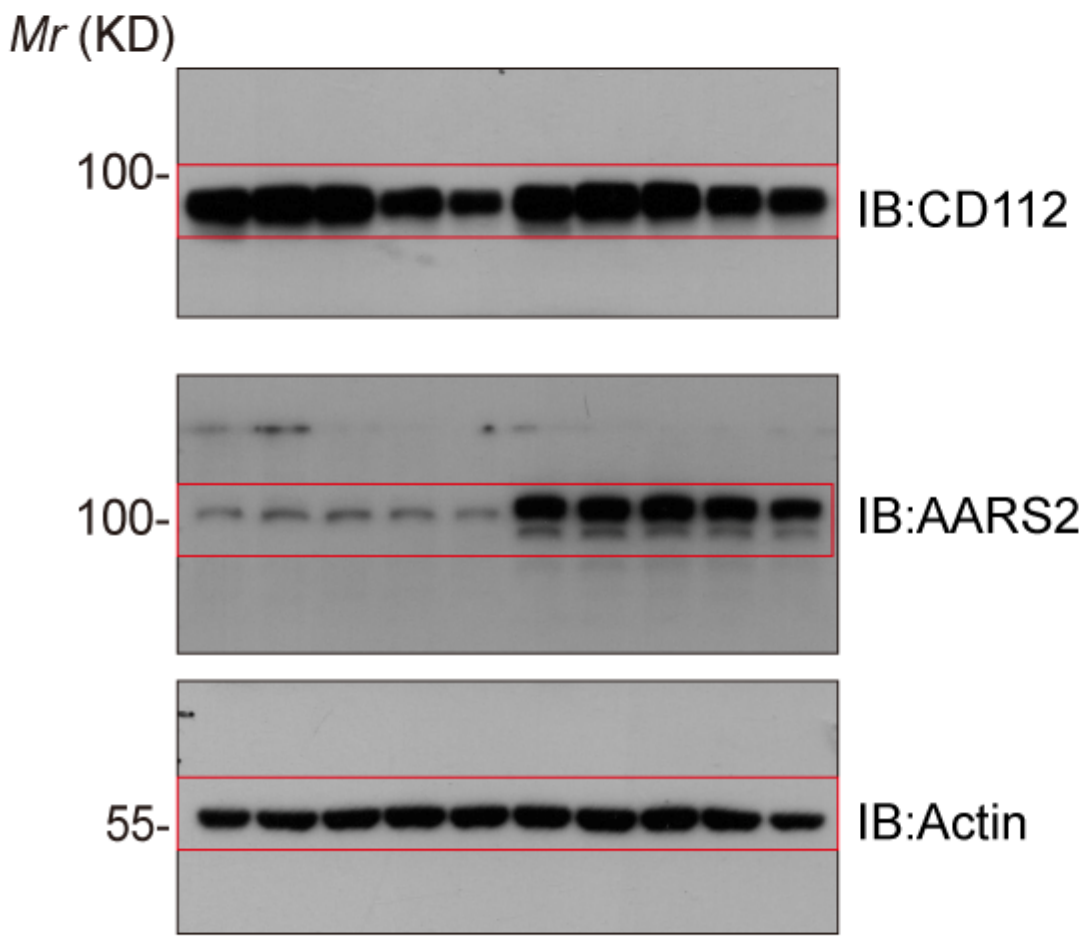

Fig. 4o

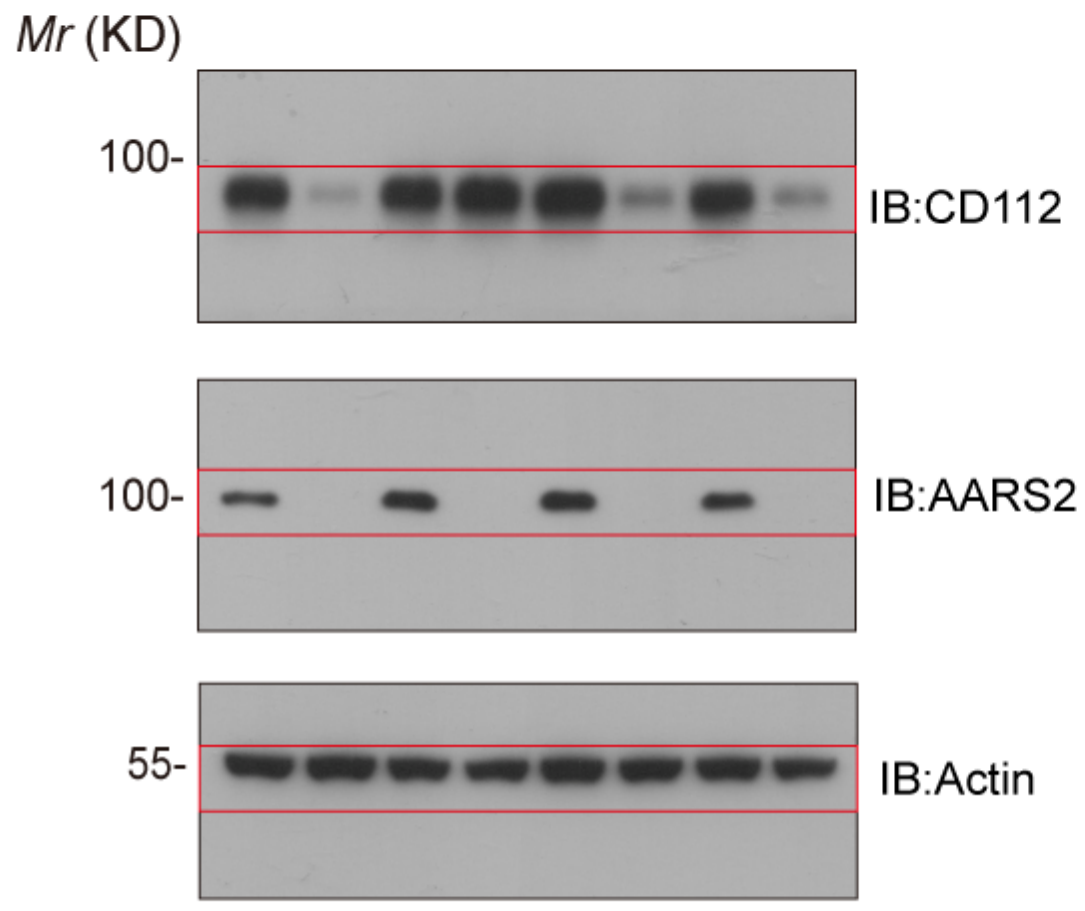

For Figure 5

Fig. 5o

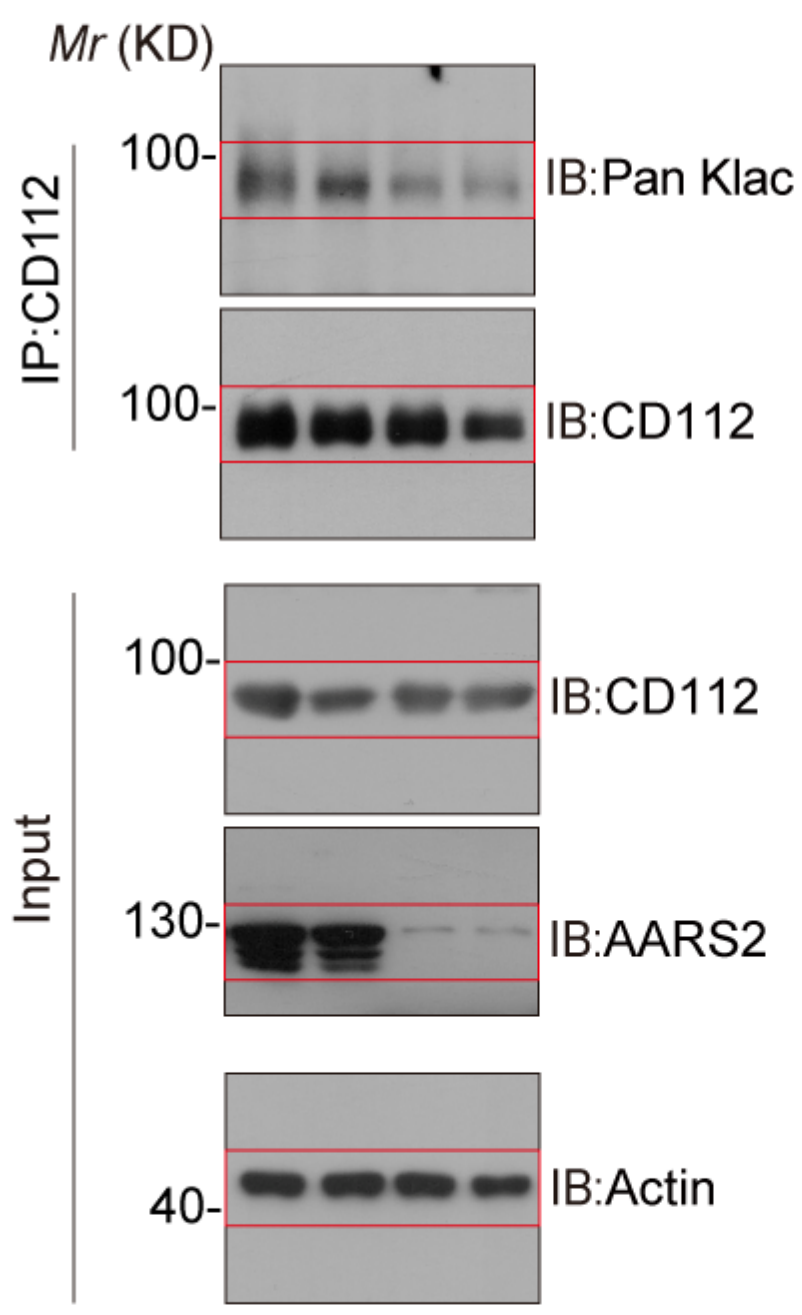

Fig. 5s

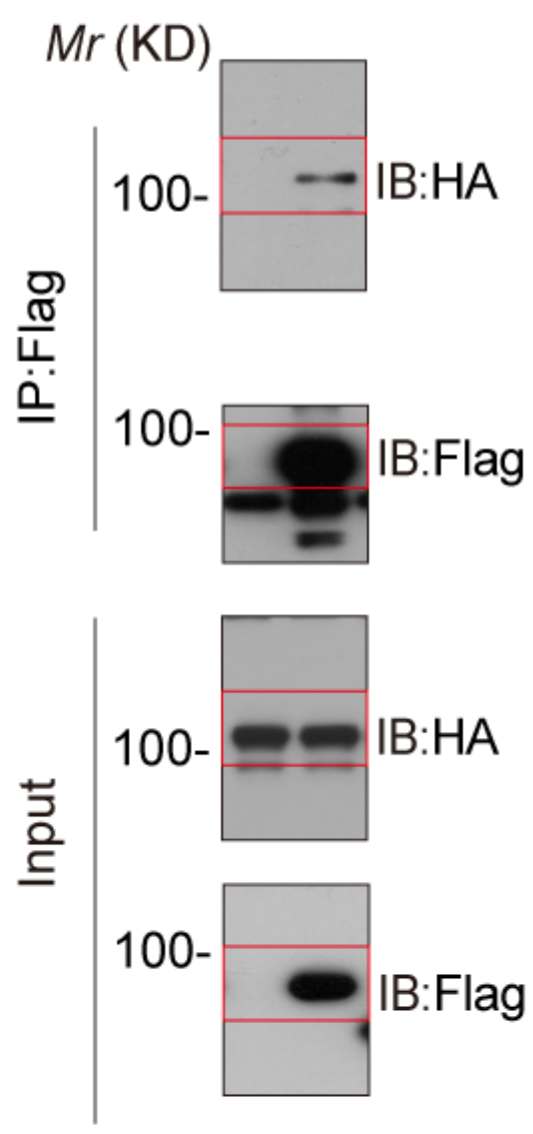

Fig. 5t

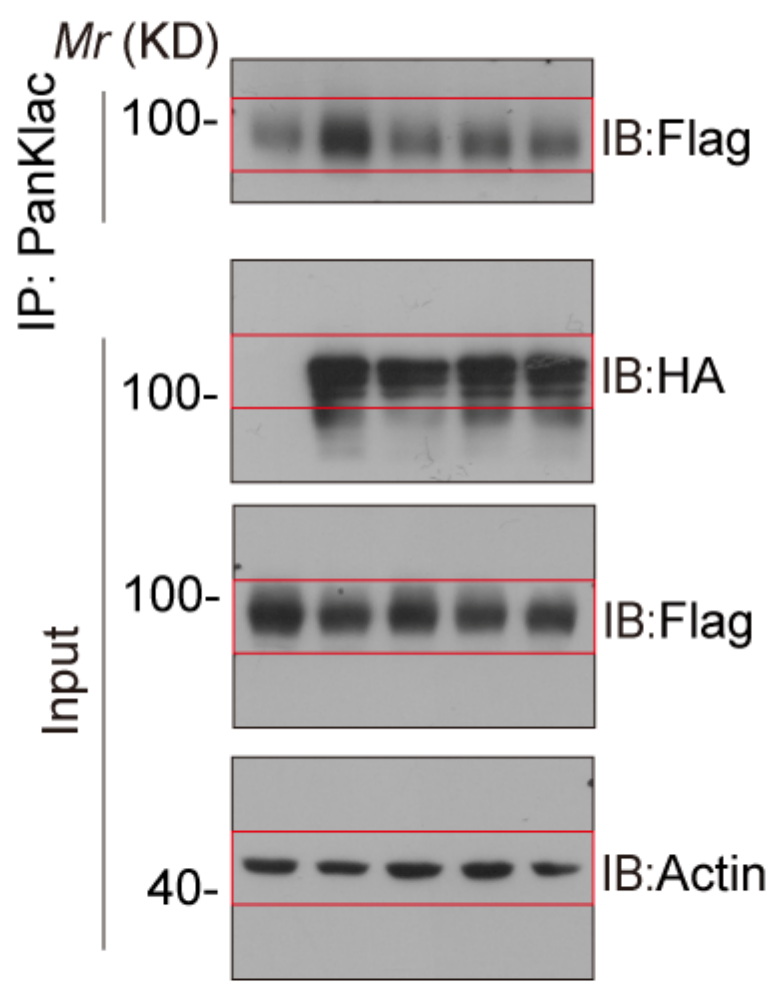

Fig. 5u

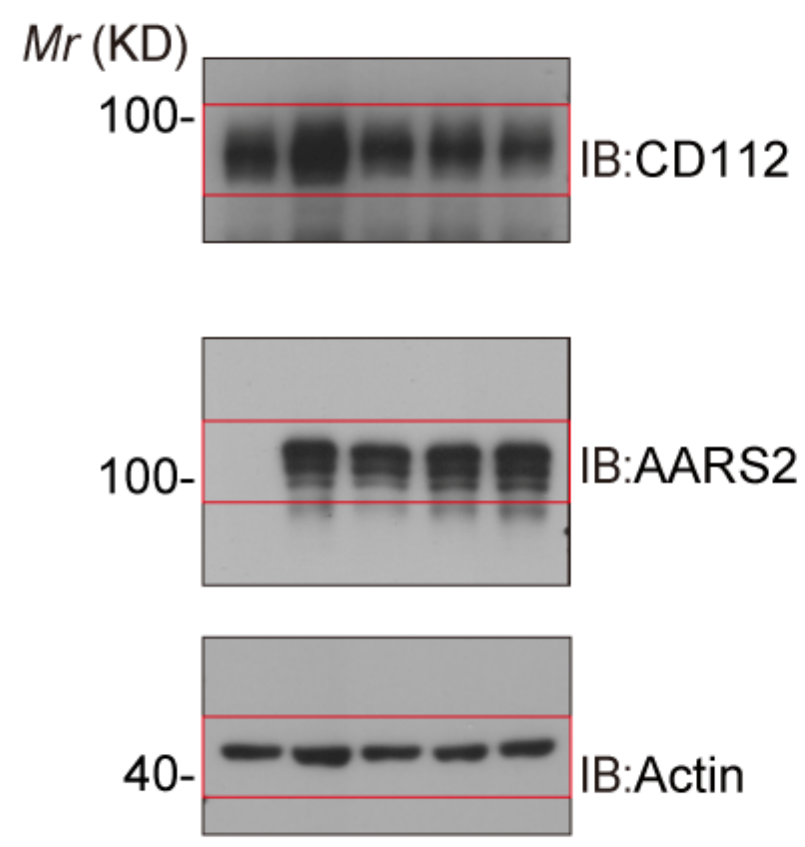

For Figure 6

Fig. 6a

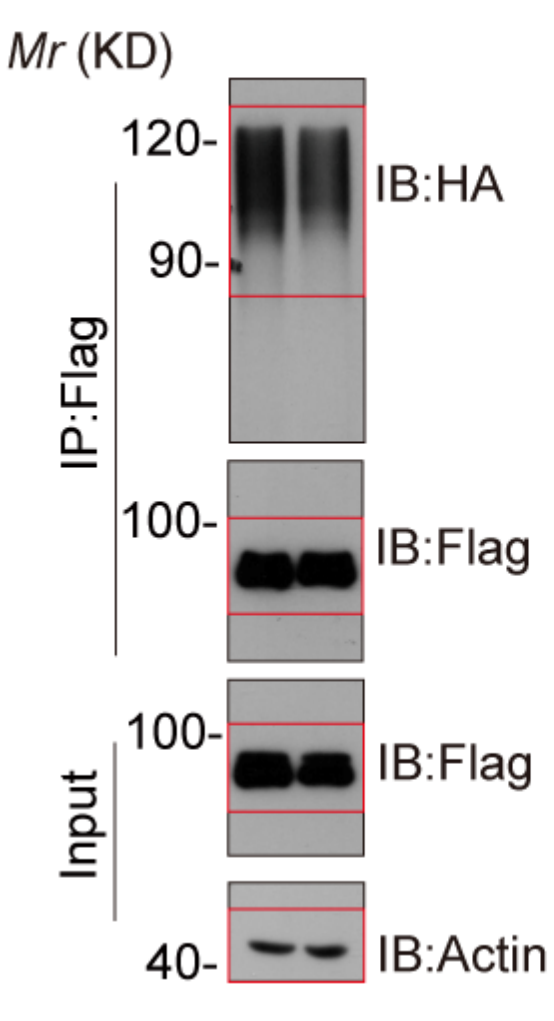

Fig. 6b

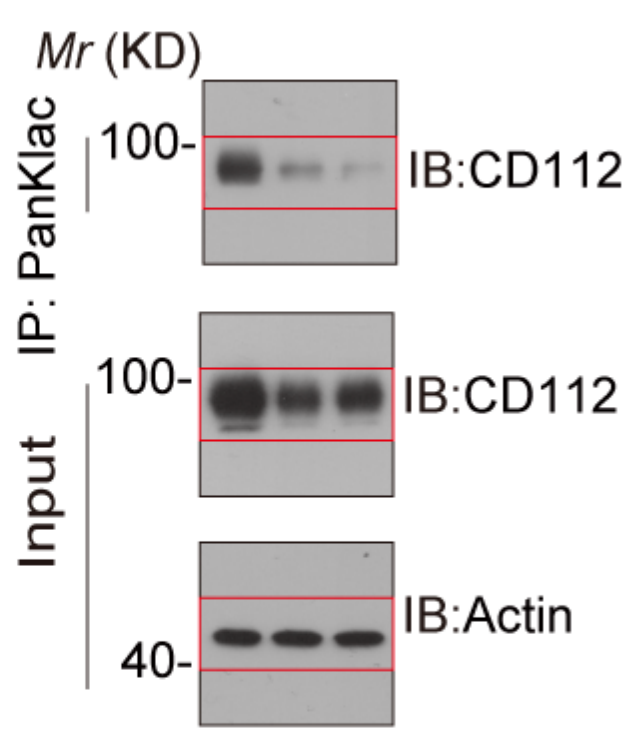

Fig. 6c

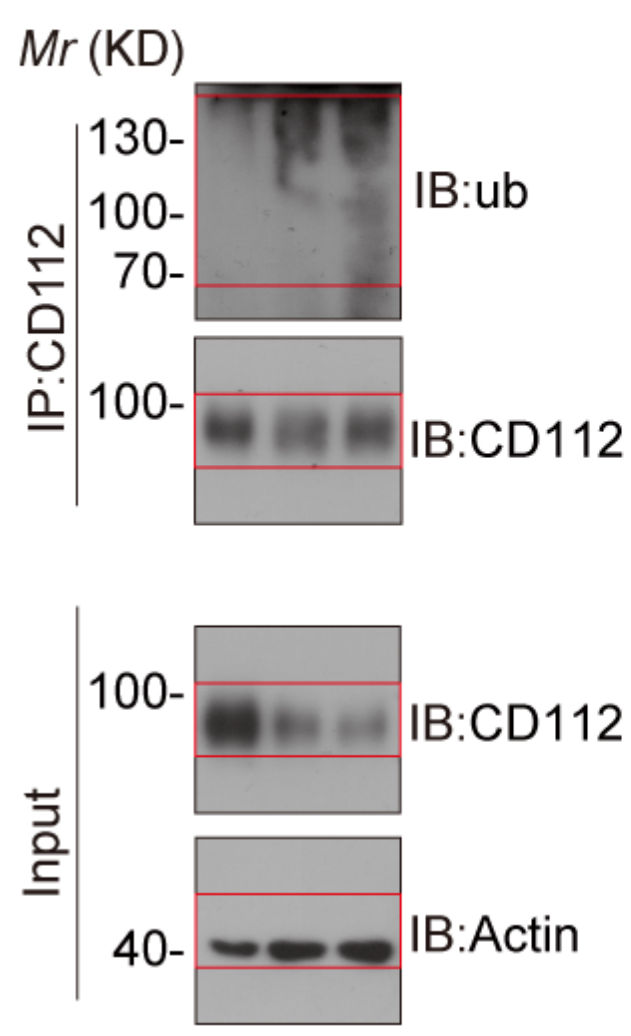

Fig. 6d

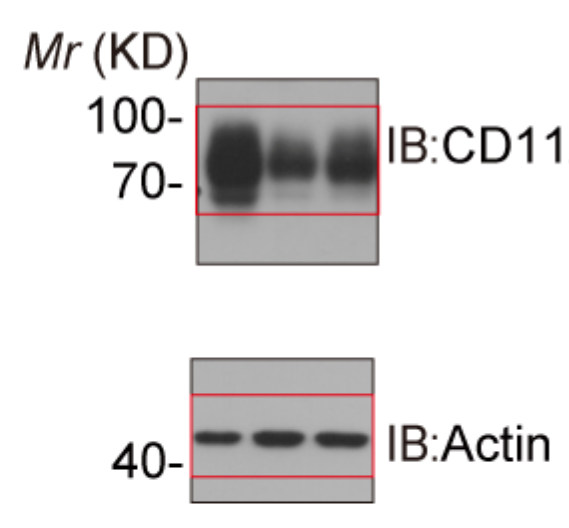

Fig. 6e

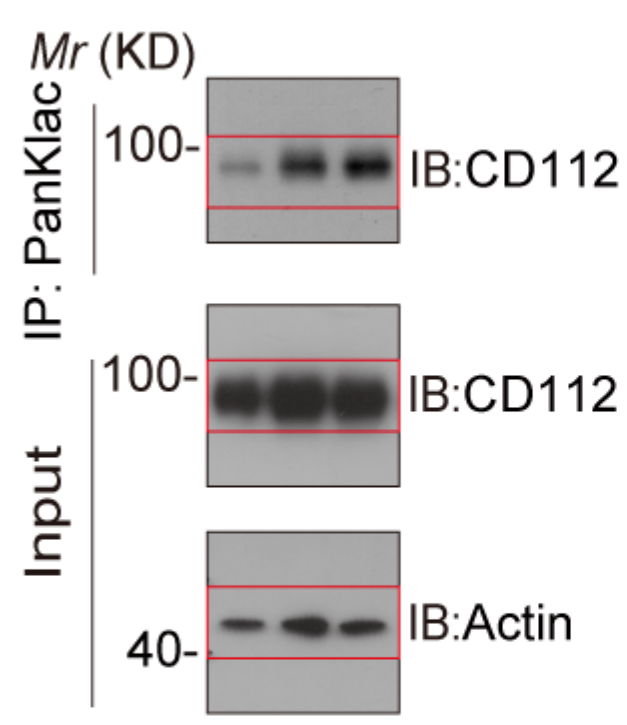

Fig. 6f

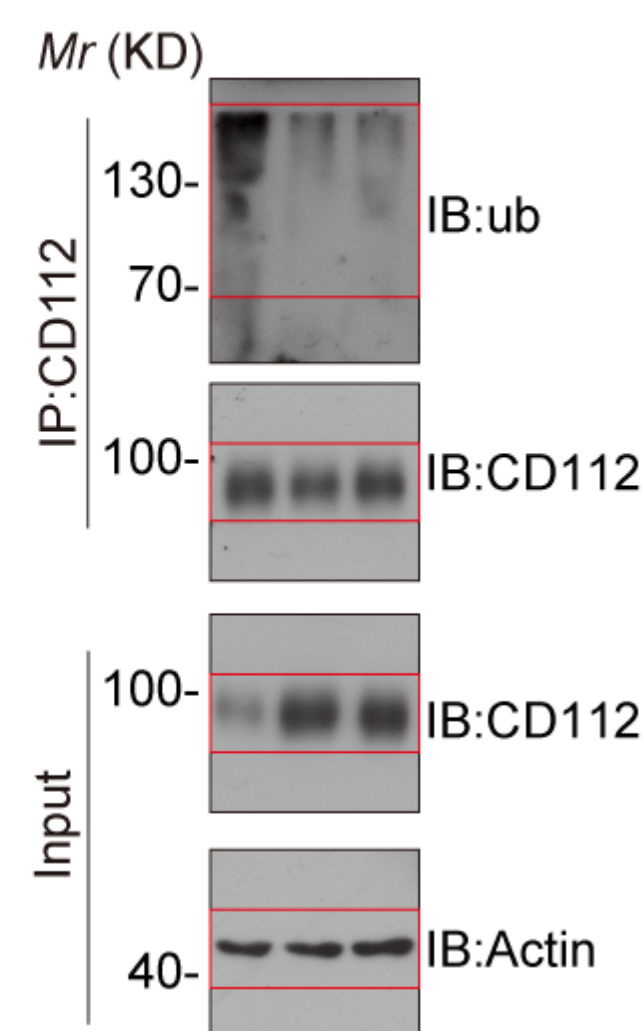

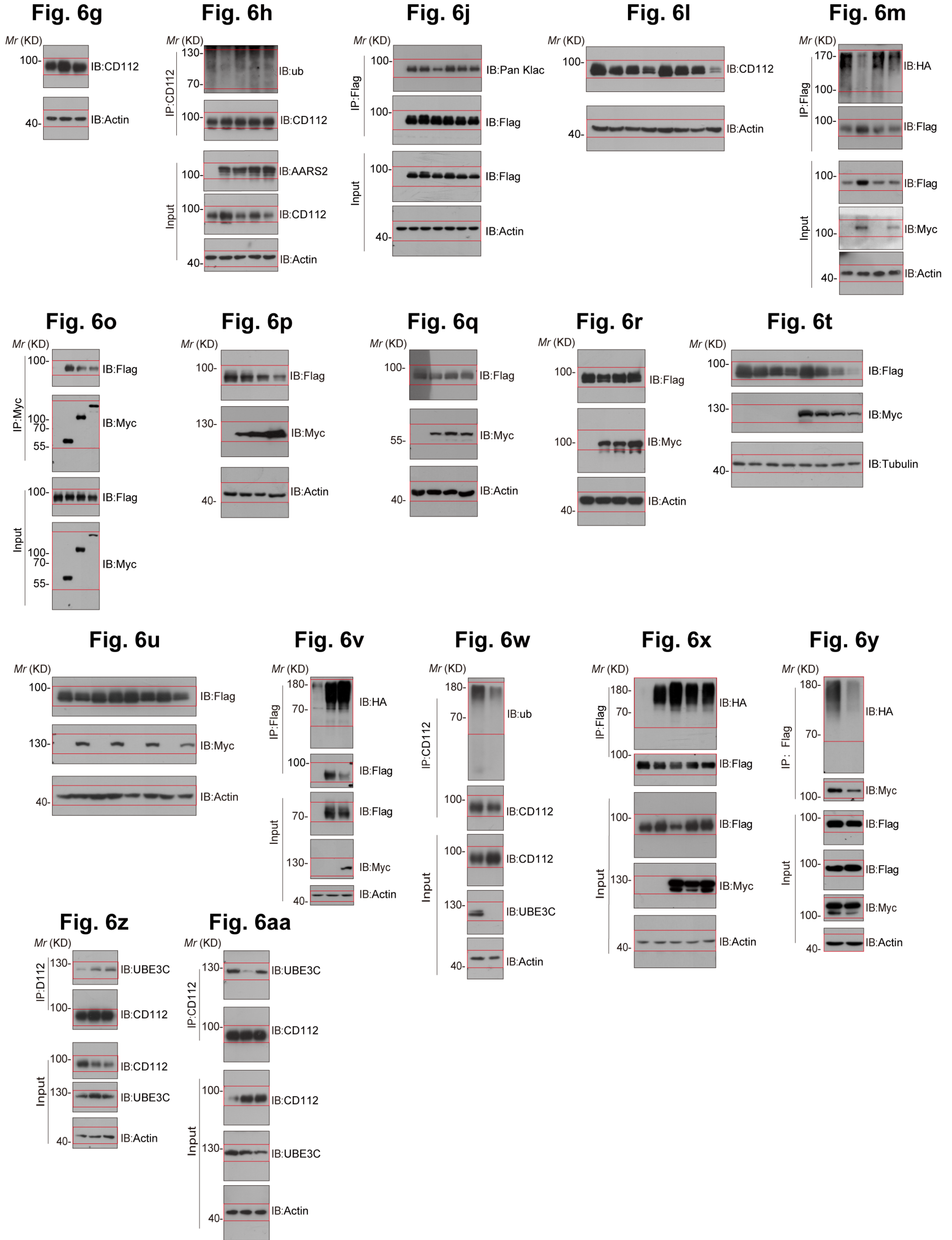

Fig. 6g
Mr (KD)
100-
IB:CD112
40-
IB:Actin
Fig. 6h
Mr (KD)
130-
70-
IB:ub
IP:CD112
100-
IB:CD112
100-
IB:AARS2
Input
100-
IB:CD112
40-
IB:Actin
Fig. 6j
Mr (KD)
100-
IB:Pan Klac
IP:Flag
100-
IB:Flag
Input
100-
IB:Flag
40-
IB:Actin
Fig. 6l
Mr (KD)
100-
IB:CD112
40-
IB:Actin
Fig. 6m
Mr (KD)
170-
100-
IB:HA
IP:Flag
100-
IB:Flag
Input
100-
IB:Flag
100-
IB:Myc
40-
IB:Actin
Fig. 6o
Mr (KD)
100-
IB:Flag
IP:Myc
100-
70-
55-
IB:Myc
Input
100-
IB:Flag
100-
70-
55-
IB:Myc
Fig. 6p
Mr (KD)
100-
IB:Flag
130-
IB:Myc
40-
IB:Actin
Fig. 6q
Mr (KD)
100-
IB:Flag
55-
IB:Myc
40-
IB:Actin
Fig. 6r
Mr (KD)
100-
IB:Flag
100-
IB:Myc
40-
IB:Actin
Fig. 6t
Mr (KD)
100-
IB:Flag
130-
IB:Myc
40-
IB:Tubulin
Fig. 6u
Mr (KD)
100-
IB:Flag
130-
IB:Myc
40-
IB:Actin
Fig. 6v
Mr (KD)
180-
70-
IB:HA
IP:Flag
100-
IB:Flag
Input
70-
IB:Flag
130-
IB:Myc
40-
IB:Actin
Fig. 6w
Mr (KD)
180-
70-
IB:ub
IP:CD112
100-
IB:CD112
Input
100-
IB:CD112
130-
IB:UBE3C
40-
IB:Actin
Fig. 6x
Mr (KD)
180-
70-
100-
IB:HA
IP:Flag
IB:Flag
Input
100-
IB:Flag
130-
IB:Myc
40-
IB:Actin
Fig. 6y
Mr (KD)
180-
70-
IB:HA
IP: Flag
100-
IB:Myc
Input
100-
IB:Flag
100-
IB:Flag
100-
IB:Myc
40-
IB:Actin
Fig. 6z
Mr (KD)
130-
IB:UBE3C
IP:D112
100-
IB:CD112
Input
100-
IB:CD112
130-
IB:UBE3C
40-
IB:Actin
Fig. 6aa
Mr (KD)
130-
IB:UBE3C
IP:CD112
100-
IB:CD112
Input
100-
IB:CD112
130-
IB:UBE3C
40-
IB:Actin

Fig. 6ab

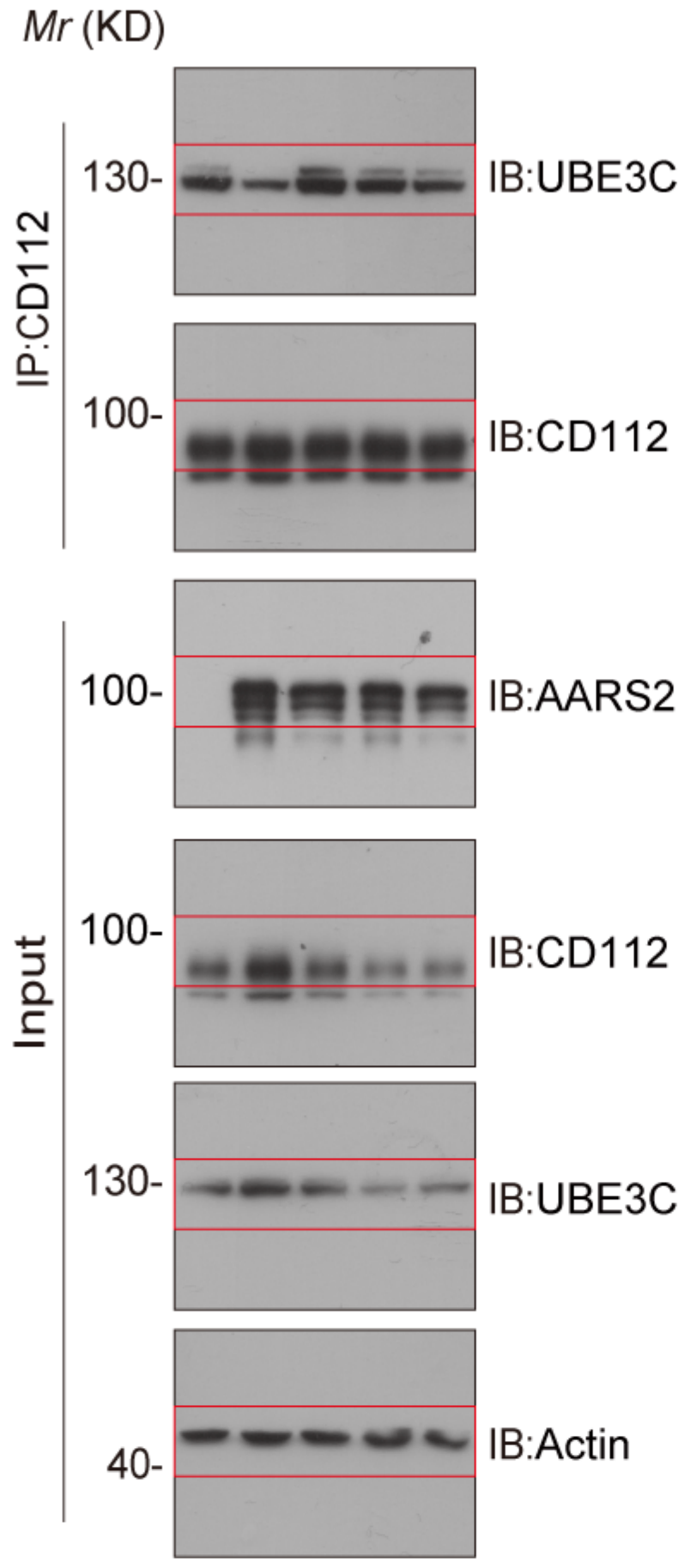

Fig. 6ac

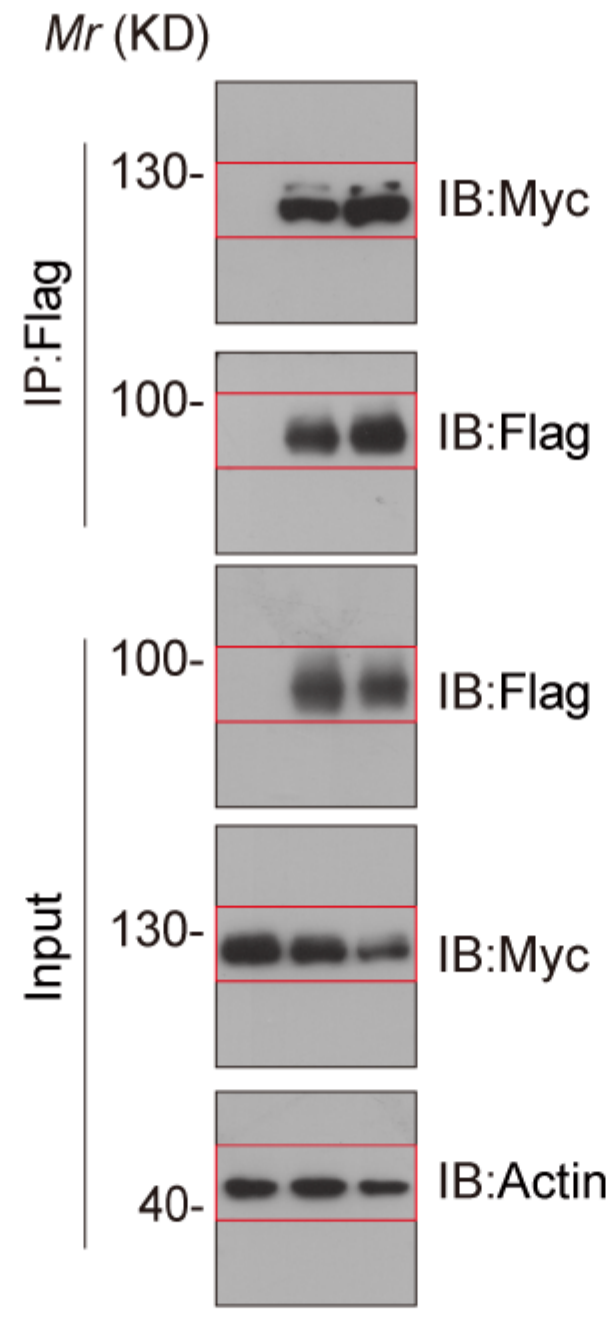

For Extended Data Figure 2

Extended Data Fig. 2a

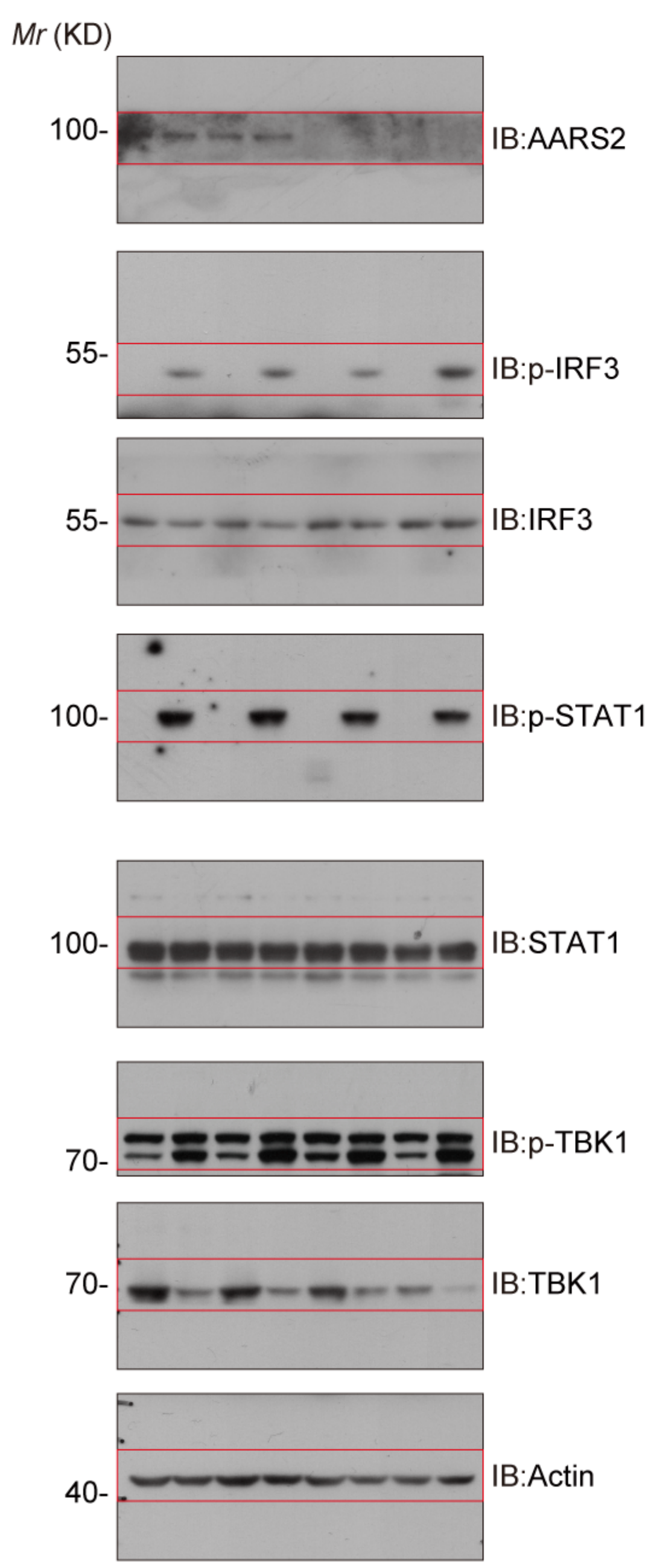

Extended Data Fig. 2b

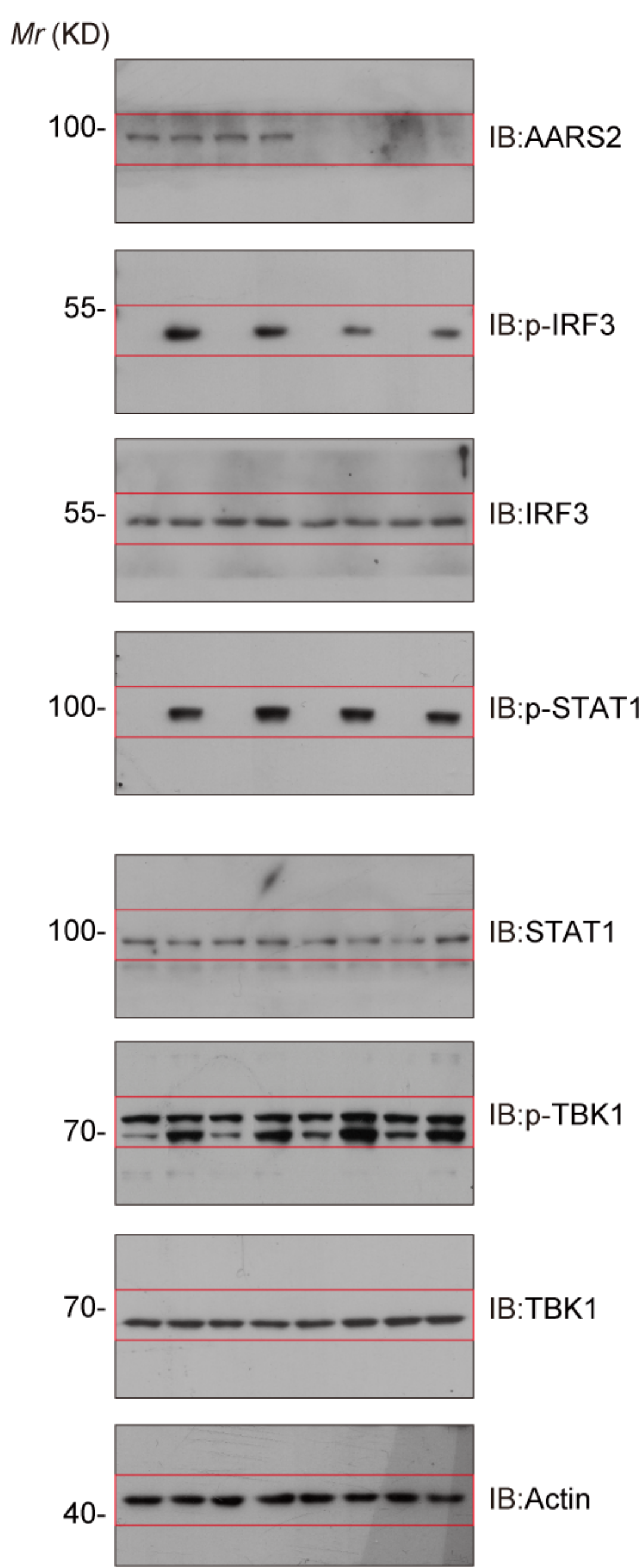

Extended Data Fig. 2c

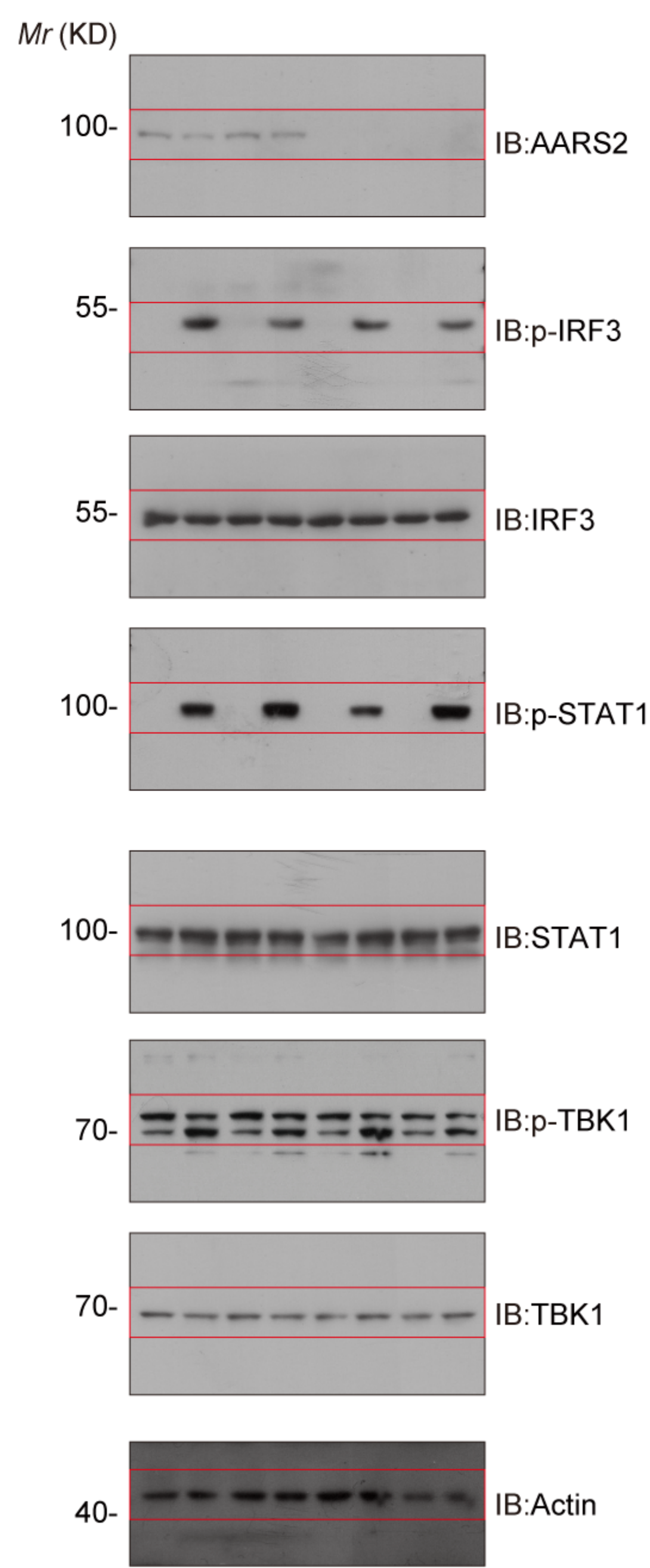

Supplement: Supplementary file 1 [file ADVS-9999-e78088-s001.pdf]
