# Supplementary material for: CD112 Lactylation Drives Dual Immune Evasion From CD8+ T Cells and NK Cells
Source: Adv Sci (Weinh). 2026 Oct 4:e78088. Online ahead of print. doi: 10.1002/advs.78088 (PMC13634482; doi:10.1002/advs.78088)
Supplement: Supplementary file 2 [file ADVS-9999-e78088-s002.docx]

**Supporting Information**

**CD112 lactylation drives dual immune evasion from CD8^+^ T cells and NK cells**

## **Extended Data Fig. 1 | AARS2 deficiency suppresses tumor growth across multiple mouse tumor models**

**a,b**, Endpoint tumor weights (a) and Kaplan–Meier survival curves (b) of C57BL/6 mice bearing control gNC, Aars1-targeting gAars1 or Aars2-targeting gAars2 B16-F10 tumors.

**c,d**, Endpoint tumor weights (c) and Kaplan–Meier survival curves (d) of C57BL/6 mice bearing control gNC, gAars1 or gAars2 EO771 tumors.

**e-h**, Effects of Aars1 or Aars2 knockout on Panc02 tumor growth in C57BL/6 mice. e, Experimental scheme. **f**, Tumor growth curves. g, Endpoint tumor weights. h, Kaplan–Meier survival curves.

**i,j**, Rescue of tumor growth by ectopic Aars2 expression in Aars2-knockout EO771 cells. Endpoint tumor weights (i) and Kaplan–Meier survival curves (j) are shown for mice bearing Aars2-knockout EO771 tumors reconstituted with empty vector or Aars2 cDNA.

**k-m**, Stereo-seq-based cell annotation of control gNC and gAars2 EO771 tumors. k, UMAP plot colored by sample group. l, UMAP plot showing major cell types, including tumor cells, CAFs, fibroblasts, macrophages, CD8+ T cells, NK cells and endothelial cells. m, UMAP plot showing detailed cell subtype annotation in the EO771 tumor microenvironment.

Data are presented as mean ± s.d. Tumor growth curves were analyzed by two-way ANOVA with multiple-comparisons test. Endpoint tumor weights were analyzed by one-way ANOVA with multiple-comparisons test or two-tailed unpaired t-test, as appropriate. Survival curves were analyzed by log-rank test. *P < 0.05, **P < 0.01; ns, not significant.

## **Extended Data Fig. 2 | AARS2 deficiency does not activate the cGAS-STING pathway in EO771 mouse tumor cells**

**a-c**, Western blot analysis of cGAS-STING pathway activation in control gNC and gAars2 tumor cells treated with HT-DNA in the presence or absence of β-alanine (a), 2-DG (b) or ARC155858 (ARC) (c). Phosphorylated and total IRF3, STAT1 and TBK1 were detected.

**d**, RNA-seq heatmap showing the expression of cGAS-STING pathway genes and interferon-stimulated genes in EO771 gAars2 versus gNC cells.

**e**, qPCR analysis of Isg56, Cxcl10 and Ifnb mRNA levels in EO771 gNC and gAars2 cells.

**f**, Proteomic heatmap showing protein levels of cGAS-STING pathway components and interferon-stimulated proteins in EO771 gAars2 versus gNC cells.

For quantified data, results are expressed as mean ± s.d. P values were determined by two-tailed unpaired t-tests. ns, not significant.

## **Extended Data Fig. 3 | Safety assessment and spatial transcriptomic annotation of TIGIT×PD-L1 bispecific antibody treatment**

**a**, Relative body weight of EO771 tumor-bearing C57BL/6 mice treated with IgG, TIGIT antibody, PD-L1 antibody or TIGIT×PD-L1 bispecific antibody (BsAb).

**b**, Peripheral blood routine analysis in the indicated treatment groups, including white blood cells (WBC), red blood cells (RBC), hemoglobin (HGB) and platelets (PLT).

**c**, Representative H&E staining images of major organs, including heart, liver, spleen, lung and kidney, from mice treated with IgG, TIGIT antibody, PD-L1 antibody or TIGIT×PD-L1 BsAb. Scale bar, 50 μm.

**d**, UMAP plot of spatial transcriptomic data from EO771 tumors treated with PD-L1 antibody or TIGIT×PD-L1 BsAb, colored by treatment group.

**e**, UMAP plot showing detailed cell subtype annotation in EO771 tumors after PD-L1 antibody or TIGIT×PD-L1 BsAb treatment.

**f**, Heatmap showing differential interaction strength between immune cells and death-high or death-low cancer cells in TIGIT×PD-L1 BsAb-treated tumors compared with PD-L1 antibody-treated tumors.

**g**, Relative body weight of humanized NCG mice bearing patient-derived xenograft tumors after treatment with IgG, TIGIT antibody, PD-L1 antibody or TIGIT×PD-L1 BsAb.

Data are presented as mean ± s.d. P values were determined by two-tailed one-way ANOVA with multiple-comparisons test or two-tailed two-way ANOVA with multiple-comparisons test, as appropriate. ns, not significant.

## **Extended Data Fig. 4 | HSV-2 oncolytic virotherapy suppresses EO771 tumor growth without evident systemic toxicity**

**a**, Experimental scheme of HSV-2 oncolytic virus treatment in C57BL/6 mice bearing control gNC or gAars2 EO771 tumors. Mice received intratumoral injection of vehicle or HSV-2.

**b,c**, Tumor growth curves (b) and Kaplan–Meier survival curves (c) of mice bearing gNC or gAars2 EO771 tumors treated with vehicle or HSV-2.

**d**, Relative body weight of mice in the indicated treatment groups during the treatment period.

**e**, Representative H&E staining images of major organs, including heart, liver, spleen, lung and kidney, from mice bearing gNC or gAars2 EO771 tumors with or without HSV-2 treatment. Scale bar, 50 μm.

**f**, Peripheral blood routine analysis in the indicated treatment groups, including white blood cells (WBC), red blood cells (RBC), hemoglobin (HGB) and platelets (PLT).

Data are presented as mean ± s.d. Tumor growth curves were analyzed by two-way ANOVA with multiple-comparisons test. Survival curves were analyzed by log-rank test. Blood routine data were analyzed by one-way ANOVA with multiple-comparisons test. *P < 0.05, **P < 0.01, ***P < 0.001, ****P < 0.0001; ns, not significant.
